# Triply Bonded Pancake π-Dimers Stabilized by Tetravalent Actinides

**DOI:** 10.1021/jacs.3c13914

**Published:** 2024-02-06

**Authors:** Luciano Barluzzi, Sean P. Ogilvie, Alan B. Dalton, Peter Kaden, Robert Gericke, Akseli Mansikkamäki, Sean R. Giblin, Richard A. Layfield

**Affiliations:** † Department of Chemistry, School of Life Sciences, University of Sussex, Brighton BN1 9QR, U.K.; ‡ Department of Physics and Astronomy, School of Mathematical and Physical Sciences, University of Sussex, Brighton BN1 9QR, U.K.; § Institute of Resource Ecology, Helmoltz-Zentrum Dresden-Rossendorf, Bautzner Landstraße 400, Dresden 01328, Germany; ∥ NMR Research Unit, University of Oulu, P.O. Box 8000, Oulu FI-90014, Finland; ⊥ School of Physics and Astronomy, Cardiff University, Cardiff CF24 3AA, U.K.

## Abstract

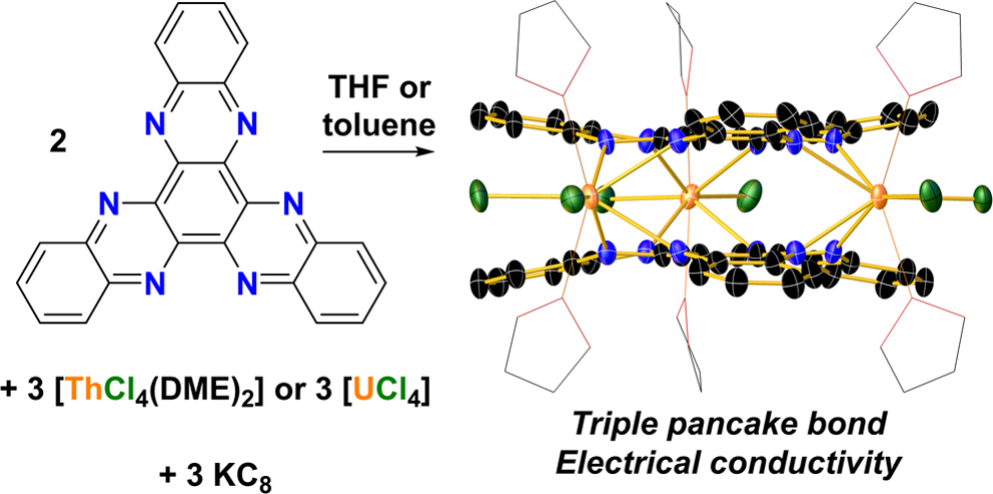

Aromatic π-stacking is a weakly attractive, noncovalent interaction often found in biological macromolecules and synthetic supramolecular chemistry. The weak nondirectional nature of π-stacking can present challenges in the design of materials owing to their weak, nondirectional nature. However, when aromatic π-systems contain an unpaired electron, stronger attraction involving face-to-face π-orbital overlap is possible, resulting in covalent so-called “pancake” bonds. Two-electron, multicenter single pancake bonds are well known, whereas four-electron double pancake bonds are rare. Higher-order pancake bonds have been predicted, but experimental systems are unknown. Here, we show that six-electron triple pancake bonds can be synthesized by a 3-fold reduction of hexaazatrinaphthylene (HAN) and subsequent stacking of the [HAN]^3–^ triradicals. Our analysis reveals a multicenter covalent triple pancake bond consisting of a σ-orbital and two equivalent π-orbitals. An electrostatic stabilizing role is established for the tetravalent thorium and uranium ions in these systems. We also show that the electronic absorption spectrum of the triple pancake bonds closely matches computational predictions, providing experimental verification of these unique interactions. The discovery of conductivity in thin films of triply bonded π-dimers presents new opportunities for the discovery of single-component molecular conductors and other spin-based molecular materials.

## Introduction

Attractive noncovalent interactions between π-electron clouds play vital roles in structural biology, drug binding^1,2^ and in the assembly of supramolecular materials.^3−5^ A different, stronger form of attraction between π-systems containing radical electrons can also occur, whereby multicenter intermolecular π-overlap leads to cofacial interactions referred to as “pancake” bonds.^6^ Pancake bonds form through covalent overlap of a singly occupied π-molecular orbital (π-SOMO) on one radical with that of another, forming a multicenter two-electron bond and a dimer with a singlet ground state.^7^ Pancake dimers display atom–overatom stacking, with molecules separated by distances shorter than the sum of van der Waals radii, in contrast to the slip-stacked structures and longer inter-ring distances found in classical π-stacks.^8−11^ Motivation for studying pancake dimers stems from their chemically modifiable electronic structure, which shows potential for applications in conducting organic materials and in materials for quantum information processing.^12−14^

**Scheme 1:**
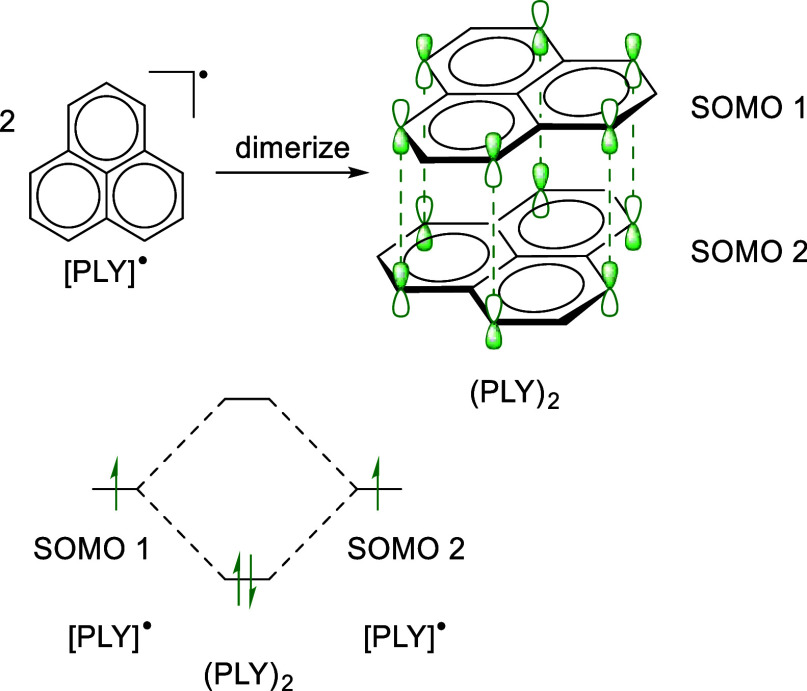
Upper: Dimerization of PLY Radicals to Give (PLY)_2_ via Cofacial π-Orbital Overlap; Lower: Simplified Molecular Orbital Scheme for (PLY)_2_

Following early studies demonstrating the electrical conductivity properties of organic π-dimers,^15−17^ attention continues to focus on pancake bonds formed through dimerization of neutral phenalenyl (PLY) radicals.^18−24^ The prototypical dimer (PLY)_2_ illustrates how SOMO–SOMO overlap results in an atom–over–atom structure and a pancake bond order of one (Scheme 1).^20^

Pancake single bonds have also been observed in dimers of electron-deficient organic compounds^25−27^ and they are frequently found in the solid-state structures of sulfur–nitrogen heterocycles.^6,28−30^ Double pancake bonds form via π-systems consisting of two unpaired electrons, as proposed for the hypothetical dimer of dithiatriazine rings (S_2_N_3_CH)_2_, thus explaining the small inter-ring separation in the experimental system (S_2_N_3_CPh)_2_.^28,29,31^ Despite challenges to the double pancake bond description,^32^ further evidence in support of such interactions has emerged.^33^ Double pancake bonding in hypothetical boron- and nitrogen-doped (PLY)_2_ dimers has also been proposed,^34^ and computational modeling of stacked dimeric triangulene graphene flakes predicts that pancake bond orders up to five might be achievable with multiradical monomers.^35^

Although pancake dimers with bond orders greater than two are unknown, the isolation of higher-order pancake bonds is a key target that would aid the validation of theoretical models while also providing new opportunities for the discovery of spin-based functional molecular materials. We now report the synthesis of triple pancake bonds based on the triradical trianion derived from the extended aromatic system hexaazatrinaphthylene, i.e., [HAN]^3–^.

## Results and Discussion

**Scheme 2:**
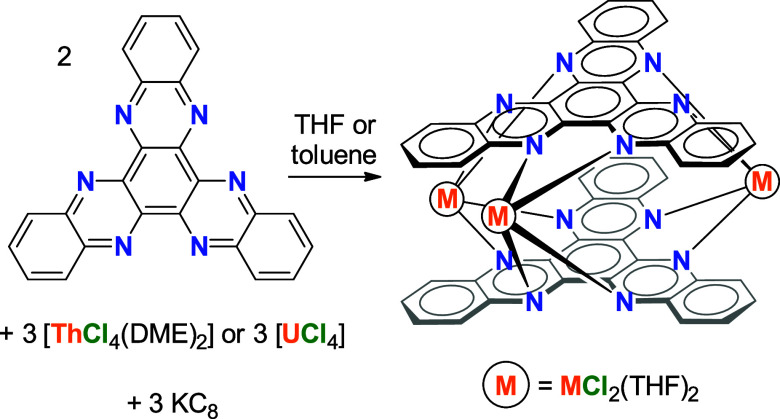
Synthesis of **1-Th** (M = Th) and **1-U** (M = U) from the Reaction of HAN with Thorium(IV) or Uranium(IV) Chloride and Potassium Graphite

To achieve effective cofacial overlap of π-orbitals in two [HAN]^3–^ anions, mitigation of Coulombic repulsions between the monomers is required. We selected the tetravalent actinides thorium(IV) and uranium(IV) for this purpose, whose large radii, high formal oxidation state, and affinity for nitrogen-donor ligands should stabilize the interaction. The target compounds [{MCl_2_(THF)_2_}_3_(HAN)_2_] (M = Th, **1-Th**; M = U, **1-U**) were synthesized by the reduction of HAN with potassium graphite (KC_8_) in the presence of the actinide(IV) precursors [ThCl_4_(DME)_2_] and UCl_4_ (Scheme 2, DME = 1,2-dimethoxyethane).

**Figure 1:**
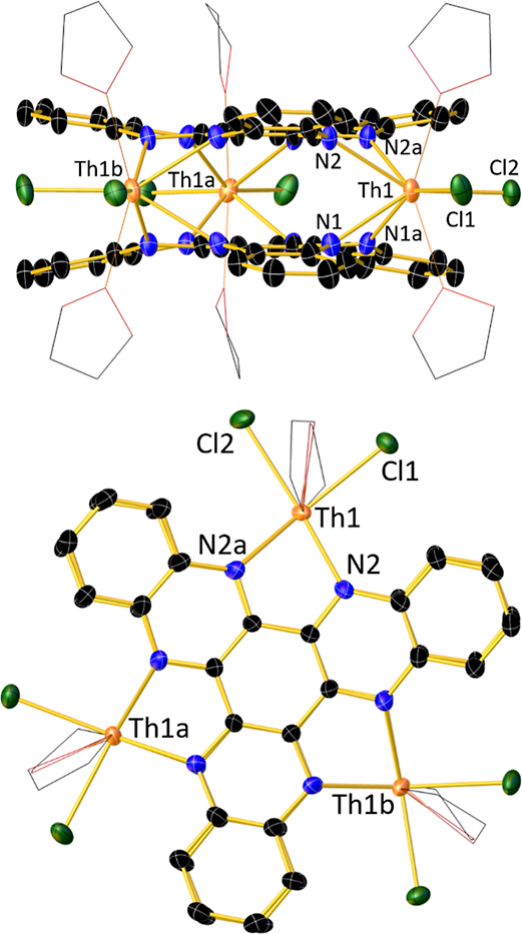
Upper: thermal ellipsoid representation (30% probability) of the molecular structure of **1-Th** viewed along the crystallographic *a*-axis (for clarity, the THF ligands are depicted as wireframes, and hydrogen atoms are omitted). Lower: molecular structure of **1-Th** viewed along the crystallographic *c*-axis.

Compound **1-Th** crystallizes in trigonal space group *R*3® with half a molecule of benzene per formula unit, and **1-U** crystallizes in monoclinic space group *P*2_1_/*m* (Table S1). Compounds **1-Th** and **1-U** have similar structures (Figures 1, S1–S3), consisting of three eight-coordinate actinide(IV) centers occupying distorted triangular dodecahedral geometries (Tables S2 and S3), with two terminal chloride ligands, two THF ligands and two bridging bidentate HAN ligands. In **1-Th**, the Th–O1 and Th–O2 bond distances are 2.571(5) and 2.595(5) Å, respectively, and the Th–Cl1 and Th–Cl2 distances are 2.686(2) and 2.693(2) Å, respectively. The Th–N1 and Th–N2 bond distances are 2.526(5) and 2.540(6) Å, respectively. In **1-U**, the average U–O bond distance is 2.58(2) Å, and the average U–Cl bond distance is 2.651(8) Å. The U–N bond distances range from 2.47(2) to 2.50(2) Å.

The concave shape of HAN in **1-Th** and **1-U** is reflected in the dihedral angles of 9.75(3) and 8.74(5)°, respectively, formed between the central and peripheral C_6_ rings, a striking feature considering that metal complexes of HAN ligands tend to be flat.^36−40^ Furthermore, the 30 atoms within one HAN ligand are eclipsed with those in the other ligand in both compounds. The vertical separations between the central C_6_ rings in **1-Th** and **1-U** are 2.86(2) and 2.829(9) Å, respectively, markedly shorter than twice the van der Waals radius of carbon (3.40 Å) and the interlayer distance of 3.35 Å in graphite.^41^ Whereas no close intermolecular contacts occur in the crystal lattice of **1-Th** (Figures S4, S5), the outer C_6_ rings of the HAN ligands in **1-U** adopt slipped supramolecular π-stacking arrangements parallel to the crystallographic *a*-axis, with C···C distances in the range 3.3–3.5 Å (Figures S6 and S7).

After **1-Th** was dried under reduced pressure, the ^1^H NMR spectrum in THF-D_8_ shows that the benzene molecules of crystallization are removed (Figures S8–S10). The ^1^H NMR spectrum of **1-Th** displays HAN resonances at chemical shifts associated with diamagnetic aromatic compounds, i.e., δ(^1^H) = 8.44–6.52 ppm. The effective magnetic moment (μ_eff_) of **1-Th** measured using the Evans NMR method is zero (Figure S11). The ^1^H NMR spectrum of **1-U** is similar to that of the thorium analog (Figures S12 and S13).

### Theoretical Study

**Figure 2:**
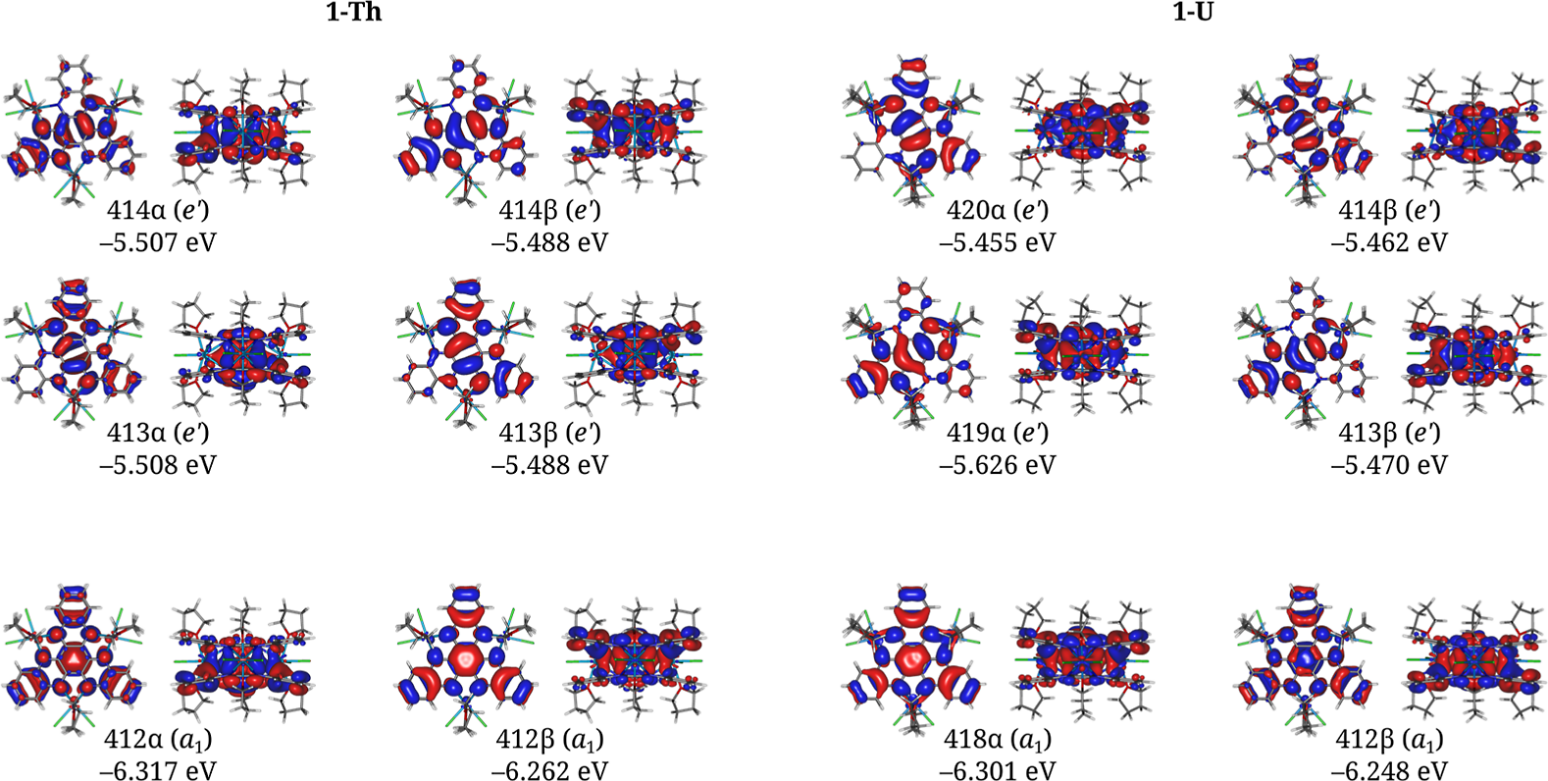
Frontier molecular orbitals in the ground spin states of **1-Th** and **1-U**. Each α- and β-orbital describing the pancake triple bonds in **1-Th** (A) and **1-U** (B) is shown top-down and side-on. Symmetry labels are based on idealized *D*_3*h*_ symmetry. The a_1_- and e′-symmetric orbitals describe the σ- and two π-components of the pancake bond, respectively.

The [HAN]^3–^ units in **1-Th** and **1-U** can occur either as a monoradical or a triradical, meaning that single or triple pancake bonding is possible in these compounds. Precisely which interaction occurs in **1-Th** and **1-U** is difficult to predict in advance of the synthesis or even in light of the concave [HAN]···[HAN] interactions found in the solid-state structures. Therefore, we investigated the bonding interactions using density functional theory (DFT) calculations. Both **1-Th** and **1-U** have similar calculated electronic structures, and to simplify the analysis we focused on **1-Th**. The three bonding orbitals between the [HAN]^3–^ radicals in **1-Th** and **1-U** in their ground spin states can be divided into a σ-bonding orbital and two π-bonding orbitals (Figure 2). Under idealized *D*_3*h*_ symmetry, the σ-bonding orbital transforms as the totally symmetric A_1_ representation and the π-bonding orbital as the E′ representation. The former is the symmetry of a conventional σ-bond, while the latter is the symmetry of a π-bond involving p-orbitals under *D*_3*h*_ symmetry.

The bonding was further studied by examining the spin-state energetics using broken-symmetry DFT (Table S4),^42−46^ the Yamaguchi projection,^47−49^ and the CAM-B3LYP exchange correlation (XC) functional.^50−52^ The geometries of the [HAN]^3–^ anions extracted from the crystal structure of **1-Th** have quartet ground states with three unpaired electrons, which are likely stabilized relative to the doublet configuration by the concave structure. The calculated energy difference between the quartet and doublet states is 382 and 712 cm^–1^ for the two radicals. The large difference in energies indicates that the ground spin state of [HAN]^3–^ is sensitive to small distortions, as reported previously.^36^ Coupling of three unpaired electrons on free [HAN]^3–^ to form a singlet ground state strongly indicates the formation of a triple bond between the radicals. The radical–radical interaction can be described as antiferromagnetic coupling between the two quartet spins. When the energy differences are described by an isotropic Heisenberg–Dirac–van Vleck Hamiltonian, i.e., *Ĥ*_HDvV_ = −*JŜ*_A_·*Ŝ*_B_ with *Ŝ*_A_ and *Ŝ*_B_ denoting effective spin operators on the [HAN]^3–^ radicals, the exchange coupling constants is *J* = −2803 cm^–1^, indicating a strong antiferromagnetic interaction that is practically a covalent bond. For comparison, the *J*-value in the dimer of 2,5,8-tri*tert*-butylphenalenyl is in the region of −1300 to −3000 cm^–1^,^53−55^ with computational values closer to the lower estimate.^20,22,56^ Thus, it is reasonable to classify the interaction between the [HAN]^3–^ anions as a covalent bond.

To further verify that the calculated exchange interaction involves three electrons on both [HAN]^3–^ anions, we also calculated the exchange coupling for a model involving only one unpaired electron on each radical. Here, the exchange coupling is massively strong with *J* = −10,739 cm^–1^. However, the spin expectation value ⟨*S*^2^⟩ is 0.973, which for two interacting SOMOs would mean minimal overlap, contradicting the *J*-value. This result implies that the interaction between the [HAN]^3–^ anions involves six electrons, all of which participate in a covalent interaction, leading to a triple bond.

To obtain a quantitative picture of the bonding energetics in **1-Th**, the molecule was partitioned into fragments and reconstructed in a stepwise manner. Three fragments were chosen: two quartet [HAN]^3–^ anions and a fragment consisting of the three [ThCl_2_(THF)_2_]^2+^ cations with a total charge of +6. The molecule was constructed from these fragments in two steps by first bonding the [HAN]^3–^ anions to each other and then bonding the resulting [(HAN)_2_]^6–^ dimer to the [ThCl_2_(THF)_2_]^2+^ cations. The energetics of the formation of **1-Th** from the fragments were studied using the Morokuma–Ziegler–Rauk energy decomposition analysis (EDA)^57−59^ and the PBE0 XC functional.^60−63^ In this approach, the molecular fragments are placed in the same geometry as in the molecule, and the energy associated with formation of the molecule from the fragments is termed the instantaneous interaction energy, Δ*E*_inst_. It is related to the bonding energy between the fragments but does not include the energy required to distort the fragments from the optimal geometries to those they possess in the final molecule. The Δ*E*_inst_ can be partitioned into electrostatic interaction Δ*E*_elstat_, orbital interaction Δ*E*_orb_, and Pauli repulsion Δ*E*_Pauli_ terms. The Δ*E*_elstat_ describes the classic electrostatic interaction between the molecular fragments before the electron densities mix, Δ*E*_orb_ describes the energy lowering once the fragment densities mix, and Δ*E*_Pauli_ describes nonclassical repulsion between the fragment densities due to the antisymmetry of the wave function. In addition, the DFT-D3 dispersion correction Δ*E*_disp_ can be separated from the other energy components. The orbital interaction energy can be further partitioned into contributions from different irreducible representations of the molecular point group. Symmetry was only utilized in the study of the bonding between the [HAN]^3–^ fragments and, due to the broken-spin nature of the fragments, the highest point-group symmetry is *C*_3*v*_. The results are given in Table 1.

**Table 1:** Energy Decomposition Analysis of **1-Th**^a^

bonding contribution		step 1	step 2
Δ*E*_inst_		1831	–11,369
Δ*E*_elstat_		1914	–10,473
Δ*E*_orb_	total	–376	–3978
	A_1_	–89	
	A_2_	–19	
	E	–268	
Δ*E*_Pauli_		432	3385
Δ*E*_disp_		–138	–303

a Energies are stated in kJ mol^–1^. Step 1 describes the interaction between two [HAN]^3–^ quartet radicals to give [(HAN)_2_]^6–^. Step 2 describes reconstruction of **1-Th** through the interaction of [(HAN)_2_]^6–^ with three [ThCl_2_(THF)_2_]^2+^ cations.

The EDA shows that the dominant interaction holding **1-Th** together is the electrostatic interaction between the [HAN]^3–^ and [ThCl_2_(THF)_2_]^2+^ fragments. Orbital interactions and dispersion make smaller but significant bonding contributions to the overall stability. The interaction between the [HAN]^3–^ anions is electrostatically strongly repulsive due to the large negative charges on the two radicals. The bonding orbital interaction describing the covalency, i.e., the pancake bonding, between the [HAN]^3–^ anions is smaller than the electrostatic repulsion but still very significant. This covalent interaction can be further divided into contributions from σ-bonding (A_1_ symmetry), π-bonding (E symmetry), and a minor component with A_2_ symmetry. Surprisingly, the π bonds appear to be the dominant covalent interaction between the [HAN]^3–^ anions, and the σ bond is about three times weaker. While the EDA results clearly support the existence of the triple pancake bond between [HAN]^3–^ anions, in terms of the overall bonding interactions in **1-Th** the pancake bond is supported by strong electrostatic and orbital (i.e., metal ligand covalency) interactions with the [ThCl_2_(THF)_2_]^2+^ cations.

### Magnetic Properties and Electrical Conductivity

In THF at 300 K, the X-band EPR spectrum of **1-Th** is featureless, confirming diamagnetic behavior (Figure S14). Although **1-U** is paramagnetic by virtue of the 5f^2^ electron configuration of uranium(IV), complexes of this species are typically EPR silent, hence the absence of an EPR signal for **1-U** is consistent with a diamagnetic [(HAN)_2_]^6–^ core (Figure S15). The electronic absorption spectra of **1-Th** and **1-U** in the UV/vis/NIR region in THF differ from those reported for complexes containing a single [HAN]^3–^ ligand.^36^ Two major absorptions occur for **1-Th** at wavelengths of 360 and 528 nm (Figure S16), which were assigned using time-dependent DFT (TD-DFT) calculations. The observed maximum at 360 nm can be associated with a set of four doubly degenerate pairs of transitions calculated to occur between 320 and 360 nm (Table S6). These transitions correspond to excitations from occupied nonbonding π-orbitals on [HAN]^3–^ and from the pancake bonding orbitals to orbitals that are antibonding with respect to the pancake bond, higher-lying combinations of [HAN]^3–^ π-orbitals, and vacant thorium 6d orbitals. The observed peak at 528 nm is probably related to a single transition predicted by TD-DFT to occur at a wavelength of 469 nm, corresponding to excitation from the pancake bonding orbitals to the pancake antibonding orbitals. Since this transition is directly related to the pancake triple bond, it should correspond to a rough estimate of the strength of the interaction. The UV/vis/NIR spectrum of **1-U** is similar, with absorbances occurring at 326 nm alongside broad absorptions spanning 500–700 nm (Figure S17). The absence of well-defined peaks in the NIR region of the spectrum of **1-U** (Figure S18) is consistent with the presence of uranium(IV).^64^

**Figure 3:**
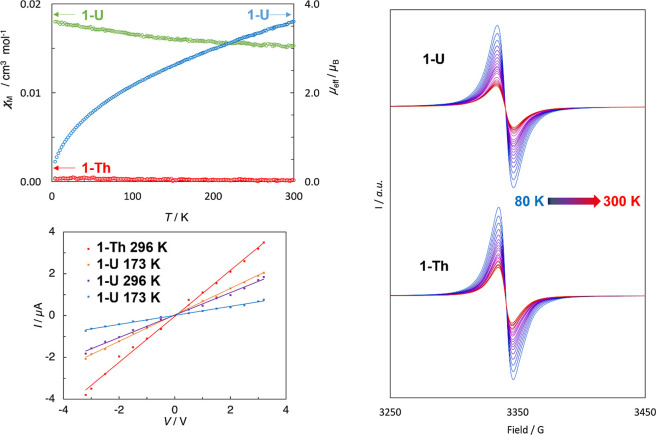
Upper left: molar magnetic susceptibility (χ_M_) as a function of temperature for **1-Th** and **1-U**, and effective magnetic moment (μ_eff_) per uranium(IV) center in **1-U**. Lower left: current–voltage characteristics for drop-cast thin films of **1-Th** and **1-U** at 296 and 173 K. Right: variable-temperature X-band EPR spectra for solid **1-Th** and **1-U** in the range 80–300 K.

The temperature dependence of the molar magnetic susceptibility (χ_M_) was measured for **1-Th** and **1-U** in the solid state at temperatures in the range of 2–300 K. The susceptibility for **1-U** is typical of uranium(IV),^65^ with χ_M_ increasing slightly from 0.016 cm^3^ mol^–1^ at 300 K to 0.019 cm^3^ mol^–1^ at 2 K, corresponding to μ_eff_ values per uranium(IV) center of 3.61 and 0.45 μ_B_, respectively (Figure 3). Compound **1-Th** unexpectedly produced a small, temperature-independent paramagnetic contribution of approximately 2.6 × 10^–4^ cm^3^ mol^–1^ (Figure 3), reminiscent of the Pauli paramagnetism in electrically conductive solids.^66,67^ The X-band EPR spectra of **1-Th** and **1-U** in the solid-state at 300 K displayed a small Lorentzian-shaped resonance centered on *g*-values of 2.0032 and 2.0036 (Figures 3, S19 and S20), respectively, accounting for approximately 2% of the sample (Figures S21–S26 and Supporting Information), and close to the free electron *g*-value. A variable-temperature EPR study at 80–300 K showed that the resonance increases in intensity with decreasing temperature, reminiscent of the conduction electron spin resonance reported for nanostructured graphite^68,69^ and graphene.^70,71^

The paramagnetism of the pancake dimers prompted us to investigate their electrical conductivity as thin films, which were prepared by drop-casting THF solutions onto interdigitated gold electrodes (Figures S27–S30). The current (*I*) was measured at 296 and 173 K using the two-probe technique, with voltages (*V*) applied at 0.5 V intervals in the range ±3.0 V and at ±3.2 V (Figure 3). Both **1-Th** and **1-U** display linear Ohmic *I*–*V* characteristics. The measured current at a given voltage is lower at 173 K. After cooling, data from repeat measurements on both compounds at 296 K were superimposable on the initial data (Figures S31 and S32). The conductivity (σ) of each material at 296 K was calculated using the resistance obtained from the *I*–*V* measurements, considering the thickness of the films, the electrode channel length, and the serpentine length along the interdigitated fingers. Values of σ = 1.72 × 10^–4^ and 0.47 × 10^–4^ S m^–1^ were determined for **1-Th** and **1-U**, respectively, similar to the conductivity reported for solution-processed semiconductor networks.^72^

The electrical conductivity of **1-Th** and **1-U** allows them to be described as single-component molecular conductors, a type of material in which charge transport often relies on noncovalent intermolecular interactions.^73,74^ For **1-U**, a conductivity mechanism is possible in which intermolecular π-stacking of the HAN ligands in the crystal lattice facilitates hopping of charge carriers, implying that nonclassical pancake and classical supramolecular π-stacks are both involved in the conductivity. The absence of intermolecular π–π stacking interactions in the lattice of **1-Th** suggests that charge carrier mobility proceeds through a different mechanism, although it is conceivable that the removal of the benzene molecules when drying the material under reduced pressure decreases the intermolecular separation, providing a potential conduction pathway.

## Conclusions

In conclusion, the reduction of HAN with KC_8_ in the presence of the tetravalent actinide chlorides [ThCl_4_(DME)_2_] and UCl_4_ results in the formation of the metal-stabilized cofacial π-dimers [{MCl_2_(THF)_2_}_3_(HAN)_2_] (M = Th, **1-Th**; M = U, **1-U**). The concave shape of the extended aromatic systems in **1-Th** and **1-U** and their orientation toward each other, with very short inter-ring separations of 2.86(2) and 2.829(9) Å, respectively, indicate the formation of covalent pancake bonds. DFT calculations reveal the presence of triple pancake bonds consisting of a σ- and two π-components. Agreement between the experimental UV/vis/NIR spectra and TD-DFT calculations support the bonding analysis. The observation of temperature-dependent EPR spectra for the notionally diamagnetic compound **1-Th** and the non-Kramers system **1-U** implied that both compounds are electrical conductors. Conductivity values derived from resistance measurements at 296 K did indeed reveal linear Ohmic *I*–*V* responses comparable to those found for solution-processed semiconductor materials. Future work on these pancake-bond materials will explore how substitution of the HAN periphery or extension of the π-conjugation impacts on the conductivity properties.

## Data Availability

Additional research data supporting this publication are available as Supporting Information at DOI: 10.25377/sussex.23703162.

## Supporting Information Available

Synthesis, spectroscopic characterization, crystallography details, magnetic measurements, EPR spectroscopy, conductivity measurements, computational details (PDF)
