# Supplementary material for: Triply Bonded Pancake π-Dimers Stabilized by Tetravalent Actinides
Source: J Am Chem Soc. 2024 Feb 6;146(6):4234–41. doi: 10.1021/jacs.3c13914 (PMC10870716; doi:10.1021/jacs.3c13914)
Supplement: Supplementary file 1 — ja3c13914_si_001.pdf [file ja3c13914_si_001.pdf]

Supplementary Information for

Triply Bonded Pancake π-Dimers Stabilized by Tetravalent Actinides

Luciano Barluzzi,[1]* Sean P. Ogilvie,[2] Alan B. Dalton,[2] Peter Kaden,[3] Robert Gericke,[3]
Akseli Mansikkamäki,[4]* Sean R. Giblin,[5] and Richard A. Layfield[1]*

[1] Department of Chemistry, School of Life Sciences, University of Sussex; Brighton, BN1 9JQ, UK.

[2] Department of Physics and Astronomy, University of Sussex; Brighton, BN1 9JQ, UK.

[3] Institute of Research Ecology, Helmoltz-Zentrum Dresden-Rossendorf (HZDR), Bautzner Landstraße 400, 01328, Dresden, Germany.

[4] NMR Research Group, University of Oulu; P.O. Box 8000, Oulu FI-90014, Finland.

[5] School of Physics and Astronomy, Cardiff University; Cardiff, CF24 3AA, UK.

l.barluzzi@sussex.ac.uk

akseli.mansikkamaki@oulu.fi

r.layfield@sussex.ac.uk

Additional research data supporting this publication are available as Supplementary Information at DOI: 10.25377/sussex.23703162.

Contents

Section		Page(s)
1	General experimental considerations	S1-S2
2	Synthesis details	S2-S4
3	FTIR spectroscopy	S5
4	X-Ray crystallography details	S6-S16
5	NMR spectroscopy	S17-S22
6	Computational details	S23-S26
7	Solution-phase EPR spectroscopy	S27-S28
8	UV-vis-NIR spectroscopy	S29-S31
9	Solid-state EPR spectroscopy	S32-S40
10	Conductivity measurements	S41-S47
11	Supplementary references	S48-S49

1. General experimental considerations

The manipulations described below were conducted under an atmosphere of high-purity argon or nitrogen with rigorous exclusion of air and water (< 0.1 ppm) using an MBraun glovebox and standard Schlenk techniques. Glassware was dried at 150°C before use. Solvents were purchased from Merck, refluxed over an appropriate drying agent for a minimum of three days (molten potassium for toluene, THF-D_8, and THF; Na/K alloy for hexane) and then distilled, degassed by three cycles of freeze-pump-thawing, and stored in ampoules over potassium mirrors (toluene, hexane) or 4 Å molecular sieves (THF-D_8, THF). Unless otherwise noted, reagents were purchased from commercial suppliers and used as received. Literature procedures were used to synthesize $ThCl_4(DME)_2$[1], hexaazatrinaphthylene (HAN)[2], KC_8[3], and UCl_4[4].

FTIR spectra were collected using a Bruker ALPHA spectrometer equipped with a Platinum ATR module in an argon-filled glovebox. NMR spectra were recorded on a Varian VNMR S400 spectrometer operating at 400 MHz (^1H frequency) and at 298 K unless otherwise stated. Chemical shifts were referenced internally to residual protons. UV/vis/NIR spectra were recorded on a Shimadzu UV-3600 Plus spectrophotometer using J-Young adapted quartz cuvettes, with baselines corrected by a blank sample of THF in a quartz cuvette.

Thorium and uranium percentage compositions of **1-Th** and **1-U** were determined using an Agilent 7500ce ICP-MS equipped with a MicroMist concentric nebulizer and a quartz double-pass spray chamber cooled to 2°C. Data were acquired in standard mode (*i.e.*, without interference removal gases) using an RF power of 1500 W. Attempts were made to obtain carbon, hydrogen and nitrogen combustion

elemental analysis. However, even with the addition of V_2O_5 the results were not satisfactory and varied even for finely ground aliquots from the same batch of material, suggesting inhomogeneous combustion.

Electron paramagnetic resonance (EPR) spectroscopy was carried out on an X-Band CW-EPR spectrometer of the ELEXSYS E500 series (Bruker Biospin, Rheinstetten, Germany) equipped with an Oxford Instruments Mercury iTC He cryostat. The measurements were recorded at 9.36 GHz in an ER 4122SHQE resonator with 0.6325 mW microwave power, 3 mT modulation amplitude and 100 kHz modulation frequency. Spin quantitation was achieved by double integration of the signal and using the spin count feature of the Bruker XEPR software, which uses the factory calibrated spatial distribution and point sample calibration factor of the resonator and measures all other factors necessary to determine the number of spins, including the quality factor Q of the specific sample.

Magnetic properties were measured on a Quantum Design Physical Property Measurement System in flame-sealed 7 mm NMR tubes. Crystalline samples were powdered before being loaded in the tube and were restrained in eicosane. Diamagnetic corrections were made with Pascal's constants [5].

Safety consideration

Natural thorium (primary isotope ^{232}Th) and depleted uranium (primary isotope ^{238}U) are weak α-emitters (4.012 MeV for natural thorium and 4.197 MeV for depleted uranium) with half-lives of 1.41×10^{10} and 4.47×10^{9} years, respectively. Manipulations and reactions should be carried out in monitored fume hoods or in an inert glovebox in a laboratory equipped with α- and β-counting equipment.

2. Synthesis details

Synthesis of [{ThCl$_2$(THF)$_2$}$_3$(HAN)$_2$] (1-Th) using a KC$_8$:HAN:Th stoichiometry of 3:1:3. Solid KC_8 (208.7 mg, 1.54 mmol) was added in portions to a stirred suspension of HAN (197.3 mg, 0.51 mmol) and $ThCl_4(DME)_2$ (855.6 mg, 1.54 mmol) in THF (20 mL). The reaction immediately developed a purple color and was then stirred at room temperature overnight. An aliquot of the reaction mixture was analyzed by ^{1}H NMR spectroscopy (Figure S8). The purple suspension was then filtered on a porosity 4 frit and the residual solids were washed with THF until the filtrate was colorless. The solvent was evaporated under reduced pressure, yielding a purple powder. Benzene (20 mL) was added to the solid and the purple suspension was filtered on a porosity 4 filter frit, and then the solid material was washed with benzene until the filtrate was colorless. The resulting purple solution was layered with hexanes and stored at room temperature for five days, resulting in dark purple crystals subsequently identified by X-ray crystallography to be **1-Th**·(0.5 C_6H_6). Isolation of the crystals followed by drying under reduced pressure led to removal of the lattice benzene and formation of **1-Th** (877.6 mg, 81%). **^{1}H NMR** (400 MHz, THF-D$_8$, δ/ppm): 8.44 (d of t, 3 H, CH,); 8.18 (d of t, 3 H, CH); 7.90 (t of d, 3 H, CH); 7.82 (t of d, 3 H, CH); 7.79 (q, 3 H, CH); 7.34 (br, s, 3H, CH); 6.67 (q, 3 H, CH); 6.52 (d, 3H, CH).

ICP-MS analysis. Compound **1-Th** (1.8 mg, 0.85 µmol) was weighed inside a glovebox, brought outside, opened and digested in fuming nitric acid (0.2 mL), giving a pale-yellow solution. A solution of concentration 0.034 µmol mL^{-1} was prepared by adding deionized water (25.0 mL). Assuming three equivalents of thorium per molecule of 1-Th, the concentration of thorium in the sample is 0.102 µmol mL^{-1} or 23.7 µg mL^{-1}. ICP-MS analysis revealed a thorium concentration of 24.0(9) µg mL^{-1}.

Attempted Synthesis of [{$ThCl_2(THF)_2$}$_3$$(HAN)_2$] (1-Th) using a KC_8:HAN:Th stoichiometry of 6:2:3. KC_8 (11.6 mg, 0.08 mmol) was added in portions to a stirred suspension of HAN (10.3 mg, 0.027 mmol) and $ThCl_4(DME)_2$ (23.6 mg, 0.04 mmol) THF-D_8 (0.7 mL). The reaction immediately developed a purple color and was then stirred at room temperature overnight. The purple suspension was then filtered into a J-Young NMR tube. The ^{1}H NMR spectrum (Figure S9) revealed the increased formation of unidentifiable side products.

Determination of the magnetic moment of 1-Th via the Evans NMR method. Compound **1-Th** (8.4 mg, 0.004 mmol) was dissolved in THF-D_8 (0.5 mL, 0.08 mol L^{-1}) and the solution was transferred to a J-Young NMR tube. A sealed glass capillary containing neat THF-D_8 was inserted into the NMR tube. The ^{1}H NMR spectrum of the reaction mixture did not reveal any shift in the solvent peak (Figure S4), confirming the diamagnetism in solution.

Synthesis of [{$UCl_2(THF)_2$}$_3$$(HAN)_2$] (1-U). Solid KC_8 (92.1 mg, 0.68 mmol) was added to a suspension of HAN (87.1 mg, 0.23 mmol) in toluene (10 mL). The reaction mixture was stirred overnight at room temperature, producing a dark powder and a colorless supernatant. The resulting suspension was added dropwise to a suspension of UCl_4 (258.7 mg, 0.68 mmol) in toluene (10 mL) and the mixture stirred at room temperature for six days, resulting in the formation of a blue supernatant and a dark solid. The supernatant was decanted. The ^{1}H NMR spectrum of the blue supernatant in THF-D_8 revealed a mixture of **1-U** and a side product (Figure S5). The solid was washed with hexane (3 × 6 mL), and then toluene (12 mL) was added to give a purple solution and a dark precipitate. The mixture was filtered onto a 0.22 µm porosity filter frit and the solid washed with toluene until the filtrate was colorless. The ^{1}H NMR spectrum of the purple extract in THF-D_8 revealed the presence of **1-U** as the only product (Figure S6). A few drops of THF were added to the toluene solution, which was then layered with hexane. Slow diffusion at room temperature yielded crystals of **1-U** (265.3 mg, 55 %). **^{1}H NMR** (400 MHz, THF-D_8, δ/ppm): 8.42 (d, 4 H, CH,); 8.17 (d, 3 H, CH); 7.90 (t, 4 H, CH); 7.83 (t, 4 H, CH); 7.35 (br, s, 3H, CH); 6.52 (d, 6H, CH).

ICP-MS analysis. Compound **1-U** (1.5 mg, 0.70 µmol) was weighed inside a glovebox, brought outside, opened and digested in fuming nitric acid (0.2 mL), giving an intense yellow solution. A solution of concentration 0.028 µmol mL^{-1} was prepared by adding deionized water (25.0 mL). Assuming three equivalents of thorium per molecule of 1-Th, the concentration of uranium in the sample is 0.085 µmol mL^{-1} or 20.1 µg mL^{-1}. ICP-MS analysis revealed a uranium concentration of 19.5(8) µg mL^{-1}.

3. FTIR spectroscopy

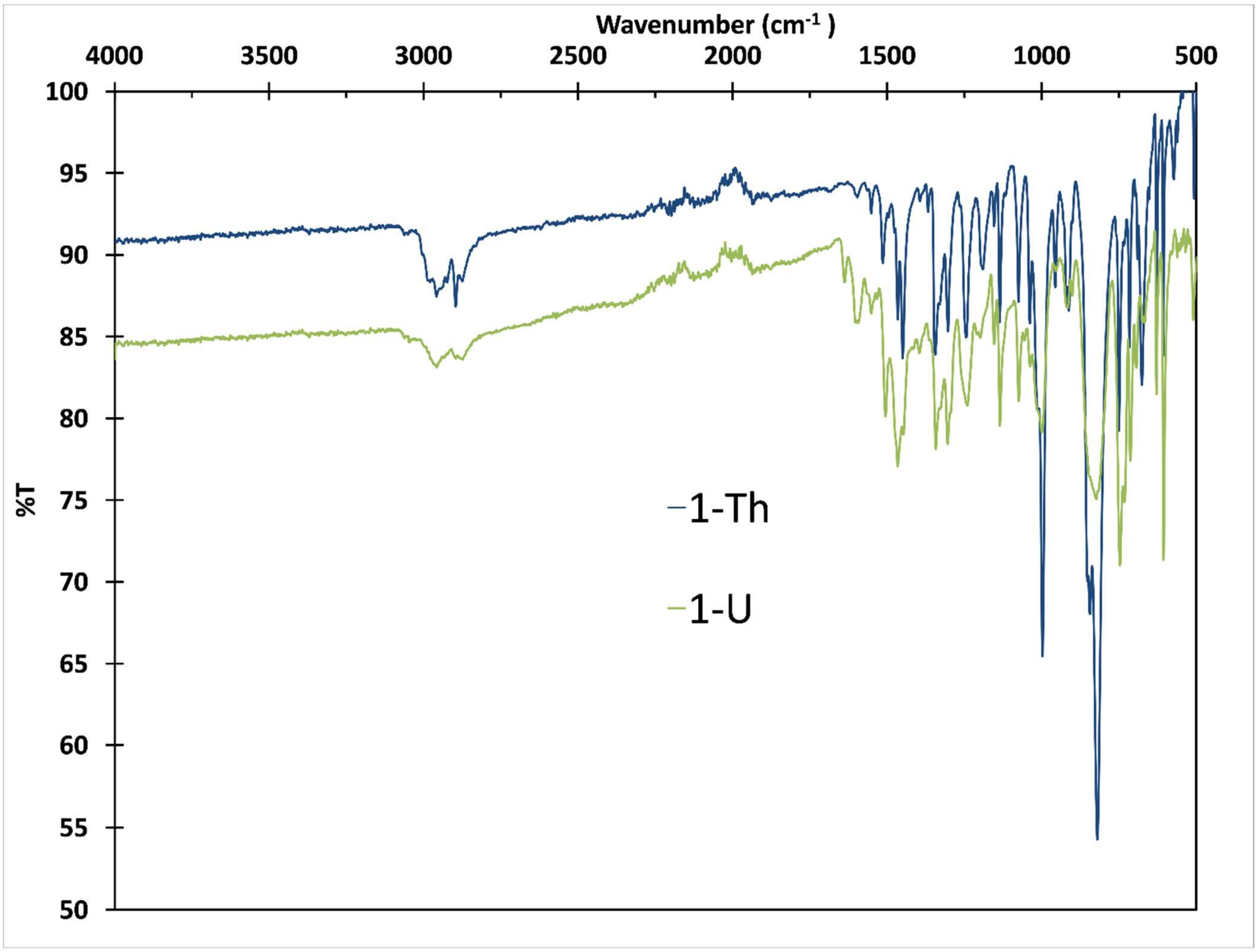

Figure S1. FTIR spectra of **1-Th** and **1-U**.

4. X-Ray crystallography details

Single-crystal X-ray diffraction measurements on **1-Th**·(0.5 C_6H_6) were carried out on an Agilent Gemini Ultra diffractometer with an Enhance Ultra (Cu-Kα) source, equipped with an Eos CCD area detector, operating in ω scanning mode to fill the Ewald sphere at 100 K. Control, integration and absorption correction were handled by the CrysAlisPro software. The crystal was mounted on a MiTeGen loop in dried Fomblin oil stored over 4 Å molecular sieves in a glovebox. Measurements on **1-U** were carried out on a Rigaku HyPix 6000HE diffractometer with a Rigaku FRE+ rotating anode (Mo-Kα) source, equipped with a UG2 goniometer and a HyPix 6000HE detector operating in ω scanning mode to fill the Ewald sphere at 100 K. The crystals were mounted on a MiTeGen MicroLoop from dried paraffin oil kept over 4 Å molecular sieves in a glovebox. Structures were solved in Olex2 [6] with SHELXT [7] using intrinsic phasing and were refined with SHELXL [8] using least squares minimization. Anisotropic thermal parameters were used for the non-hydrogen atoms and isotropic parameters for the hydrogen atoms. Hydrogen atoms were added geometrically and refined using a riding model. Crystallographic data and refinement details are given in Table S1. A multi-scan absorption correction was applied on crystals of **1-U**. An empirical absorption correction using spherical harmonics was applied, implemented in the SCALE3 ABSPACK scaling algorithm. Crystals of **1-U** were twinned and were treated using a two-component twin law, which rotates component 2 by 179.9904 degrees along the [-0.00 -0.00 1.00] reciprocal direction. The two scales of the twin components are 0.59615 and 0.40385 respectively.

Table S1. Crystal data and structural refinement for **1-Th**·(0.5 C_6H_6) and **1-U**.

	**1-Th**·(0.5 C_6H_6)	**1-U**
CCDC ref. code	2298029	2298030
Formula	$C_{75}H_{75}Cl_6N_{12}O_6Th_3$	$C_{72}H_{72}Cl_6N_{12}O_6U_3$
Crystal size (mm)	0.4 × 0.3 × 0.2	0.16 × 0.09 × 0.08
Crystal System	trigonal	monoclinic
Space Group	$R\bar{3}$	$P2_1/m$
Volume (Å^3)	11002.0(3)	3899.53(16)
a (Å)	24.9587(4)	13.5383(4)
b (Å)	24.9587(4)	18.3183(4)
c (Å)	20.3937(4)	15.7608(3)
α (°)	90	90
β (°)	90	93.917(2)
γ (°)	120	90
Z	6	2
Formula Weight	2149.29	2128.2
Density (g cm^{-3})	1.946	1.813
μ (mm^{-1})	21.931	19.688
F(000)	6174	2028
Temperature (K)	99.8(10)	100.0(1)
Total Reflections	22974	22529
Unique Reflections	4751	22529
R_{int}	0.0871	-
R Indices [$I > 2\sigma(I)$]	$R_1 = 0.0356$	$R_1 = 0.1084$
	$wR_2 = 0.0820$	$wR_2 = 0.2875$
Largest Diff. Peak and Hole (e.A^{-3})	1.89 and –1.47	5.75 and –3.57
GOF	1.001	1.228

Detailed structural description of 1-Th

Compound **1-Th** crystallizes with 0.5 molecules of benzene per formula unit in the lattice in the rhombohedral space group $R\overline{3}$. The asymmetric unit exhibits a three-fold symmetry and comprises one third of the molecule. Each thorium center is eight-coordinate and occupies a distorted triangular dodecahedral geometry, and is bound by two THF ligands, two chloride ligands, and four nitrogen atoms from two HAN ligands. The Th–O1 and Th–O2 bond distances are 2.571(5) and 2.595(5) Å, respectively, and the Th–Cl1 and Th–Cl2 distances are 2.686(2) and 2.693(2) Å, respectively. The Th–N1 and Th – N2 bond distances of 2.526(5) and 2.540(6) Å, respectively, are similar to those in thorium complexes with neutral bipyridine ligands, i.e., approximately 2.6 Å [9] The central C_6 rings on the HAN ligands, the pyrazine $\{C_4N_2\}$ rings, and the peripheral C_6 rings are individually planar, but the HAN ligands overall bend appreciably into concave shapes. The dihedral angle between the central C_6 rings and their respective peripheral C_6 rings is 9.75(3)°. The C–C bond distances in the HAN ligands are all similar, ranging from 1.38(1) Å to 1.427(8) Å.

Detailed structural description of 1-U

Complex **1-U** crystallizes in the monoclinic space group $P2_1/m$. The asymmetric unit exhibits a two-fold symmetry and comprises half of the molecule. Each uranium center is eight-coordinate and occupies a distorted triangular dodecahedral geometry, and is bound by two THF ligands, two chloride ligands, and four nitrogen atoms from two HAN ligands. The average U–O bond distance is 2.58(2) Å, and the average U–Cl bond distance is 2.651(8) Å. The U–N bond distances range from 2.47(2) to 2.50(2) Å and are close to those found in U(IV) complexes with neutral bipyridine ligands, i.e., approximately 2.55 Å [10]. The structural properties of the constituent six-membered rings in **1-U** are essentially the same as in **1-Th**, with the dihedral angle between the central C_6 rings and their respective peripheral C_6 rings being 8.74(5)°. The C–C bond distances in the HAN ligands range from 1.36(3) Å to 1.46(3) Å, consistent with no loss of aromaticity. As in **1-Th**, the absence of geometric distortions in terms of C–C bond distances in **1-U** are consistent with pairing of the additional three electrons per $[HAN]^{3-}$ ligand in a pancake triple bond.

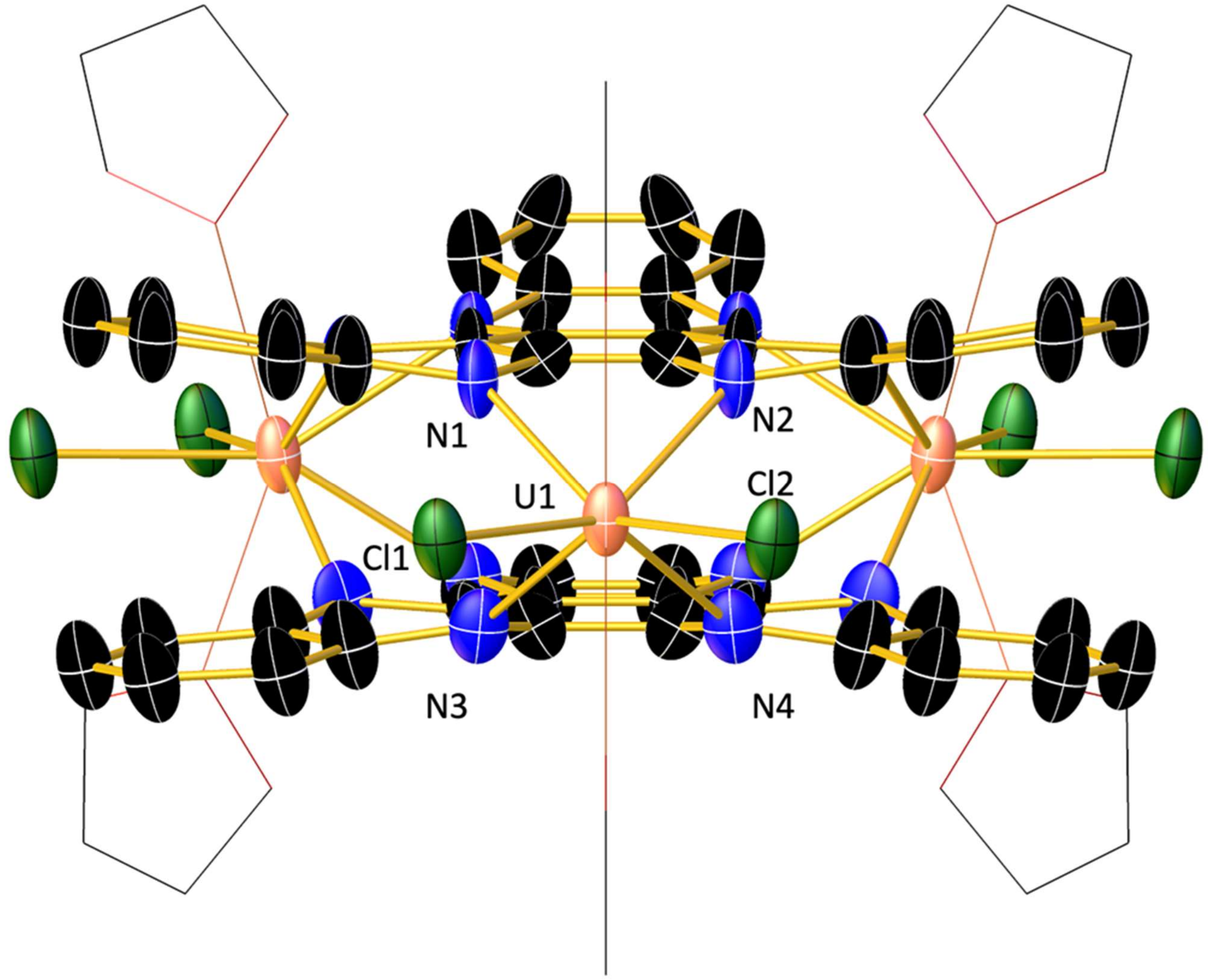

Figure S2. Molecular structure of **1-U** viewed along the crystallographic *c*-axis. Thermal ellipsoids at 30% probability. For clarity, the hydrogen atoms are not shown and the THF ligands are depicted in wireframe.

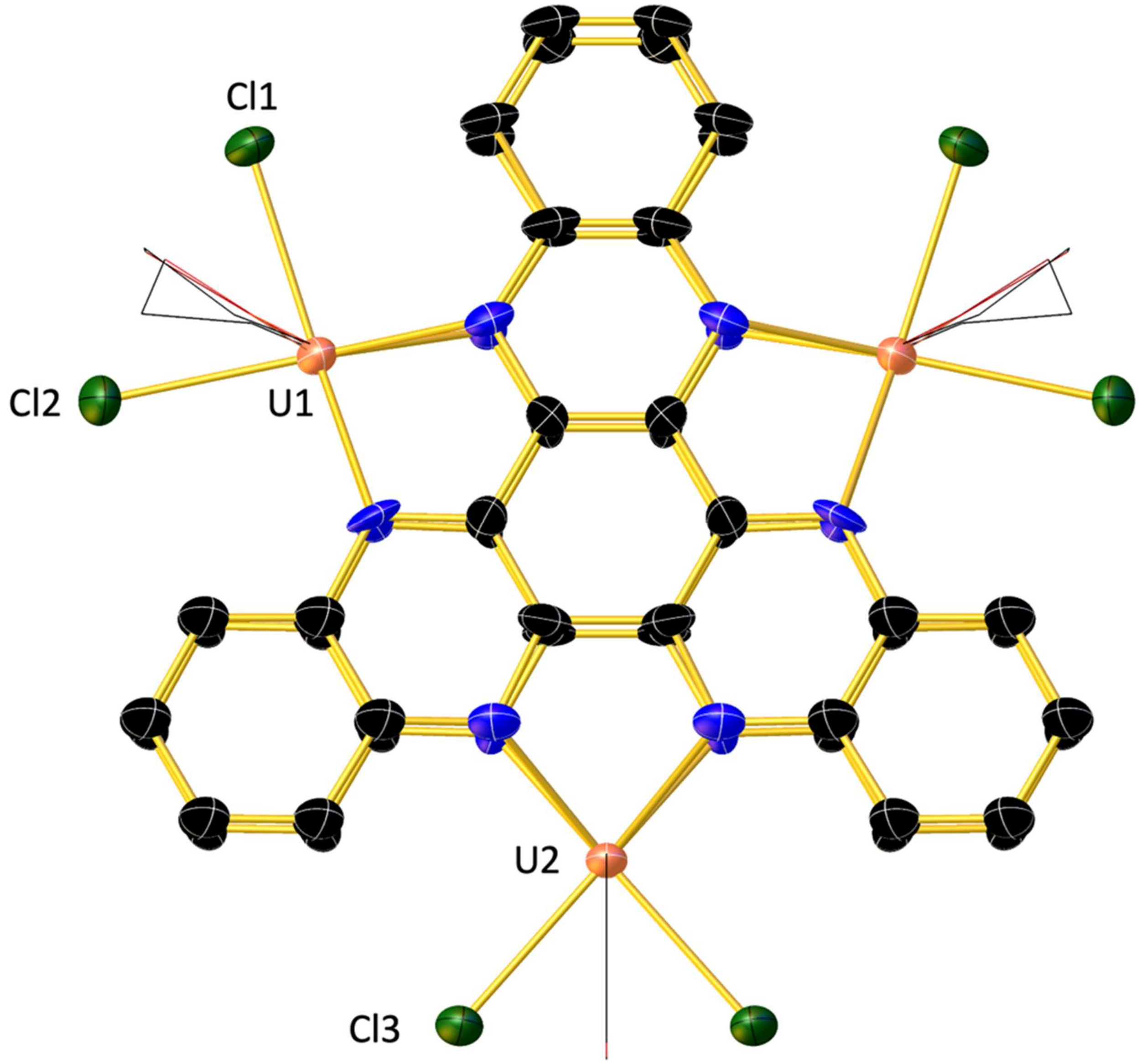

Figure S3. Molecular structure of 1-U viewed along the crystallographic *a*-axis. Thermal ellipsoids at 30% probability. For clarity, the hydrogen atoms are not shown and the THF ligands are depicted in wireframe.

Table S2. SHAPE analysis of the thorium coordination geometry in **1-Th**.

SHAPE Geometry (Code)	Th1
Octagon (OP-8)	30.300
Heptagonal Pyramid (HPY-8)	23.140
Hexagonal Bipyramid (HBPY-8)	15.845
Cube (CU-8)	12.594
Square Antiprism (SAPR-8)	4.962
Triangular Dodecahedron (TDD-8)	2.947
Johnson Gyrobifastigium J26 (JGBF-8)	15.844
Johnson Elongated Triangular Bipyramid J14 (JETBPY-8)	28.823
Biaugmented Trigonal Prism J50 (JBTPR-8)	4.131
Biaugmented Trigonal Prism (BTPR-8)	3.686
Snub Disphenoid J84 (JSD-8)	5.907
Triakis Tetrahedron (TT-8)	13.119
Elongated Trigonal Pyramid (ETBPY-8)	24.520

Table S3. SHAPE analysis of the uranium coordination geometry in **1-U**.

SHAPE Geometry (Code)	U1
Octagon (OP-8)	30.951
Heptagonal Pyramid (HPY-8)	23.134
Hexagonal Bipyramid (HBPY-8)	15.463
Cube (CU-8)	12.012
Square Antiprism (SAPR-8)	4.427
Triangular Dodecahedron (TDD-8)	2.540
Johnson Gyrobifastigium J26 (JGBF-8)	15.721
Johnson Elongated Triangular Bipyramid J14 (JETBPY-8)	29.029
Biaugmented Trigonal Prism J50 (JBTPR-8)	3.655
Biaugmented Trigonal Prism (BTPR-8)	3.236
Snub Disphenoid J84 (JSD-8)	5.461
Triakis Tetrahedron (TT-8)	12.640
Elongated Trigonal Pyramid (ETBPY-8)	24.634

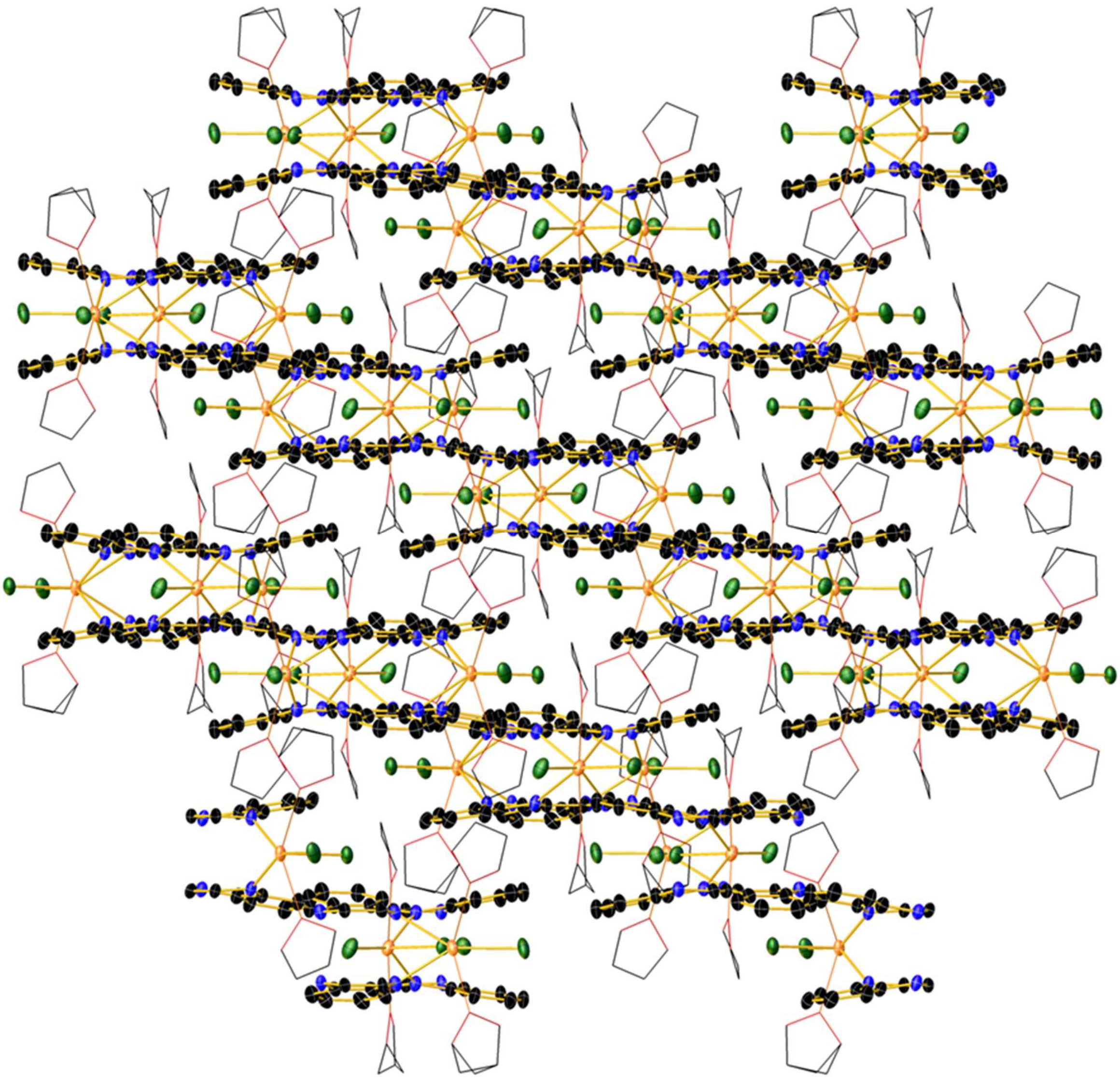

Figure S4. Packing in the crystal lattice of **1-Th** viewed along the crystallographic *a*-axis. Thermal ellipsoids at 30% probability. For clarity, the hydrogen atoms are not shown.

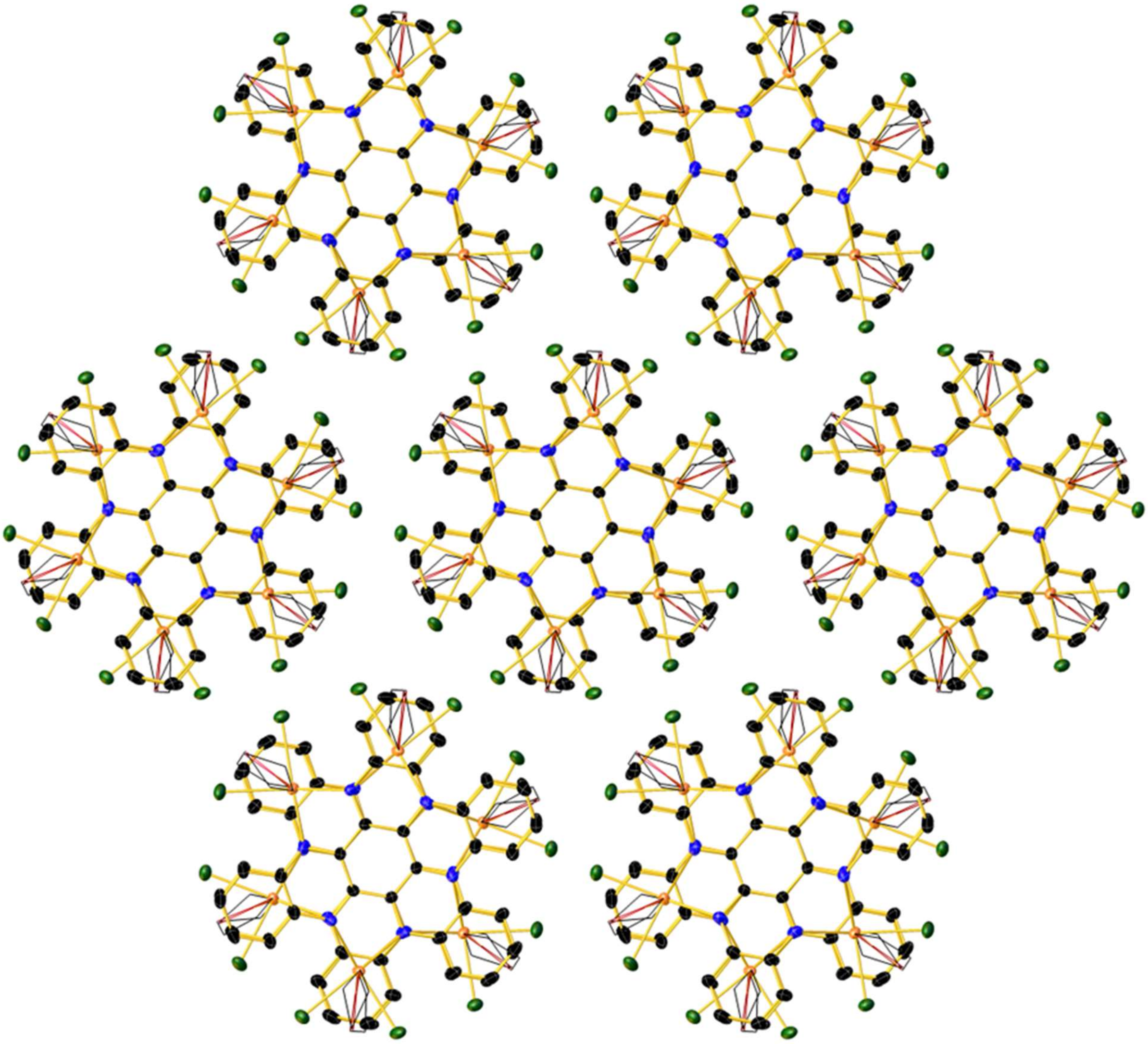

Figure S5. Packing in the crystal lattice of **1-Th** viewed along the crystallographic *c*-axis. Thermal ellipsoids at 30% probability. For clarity, the hydrogen atoms are not shown.

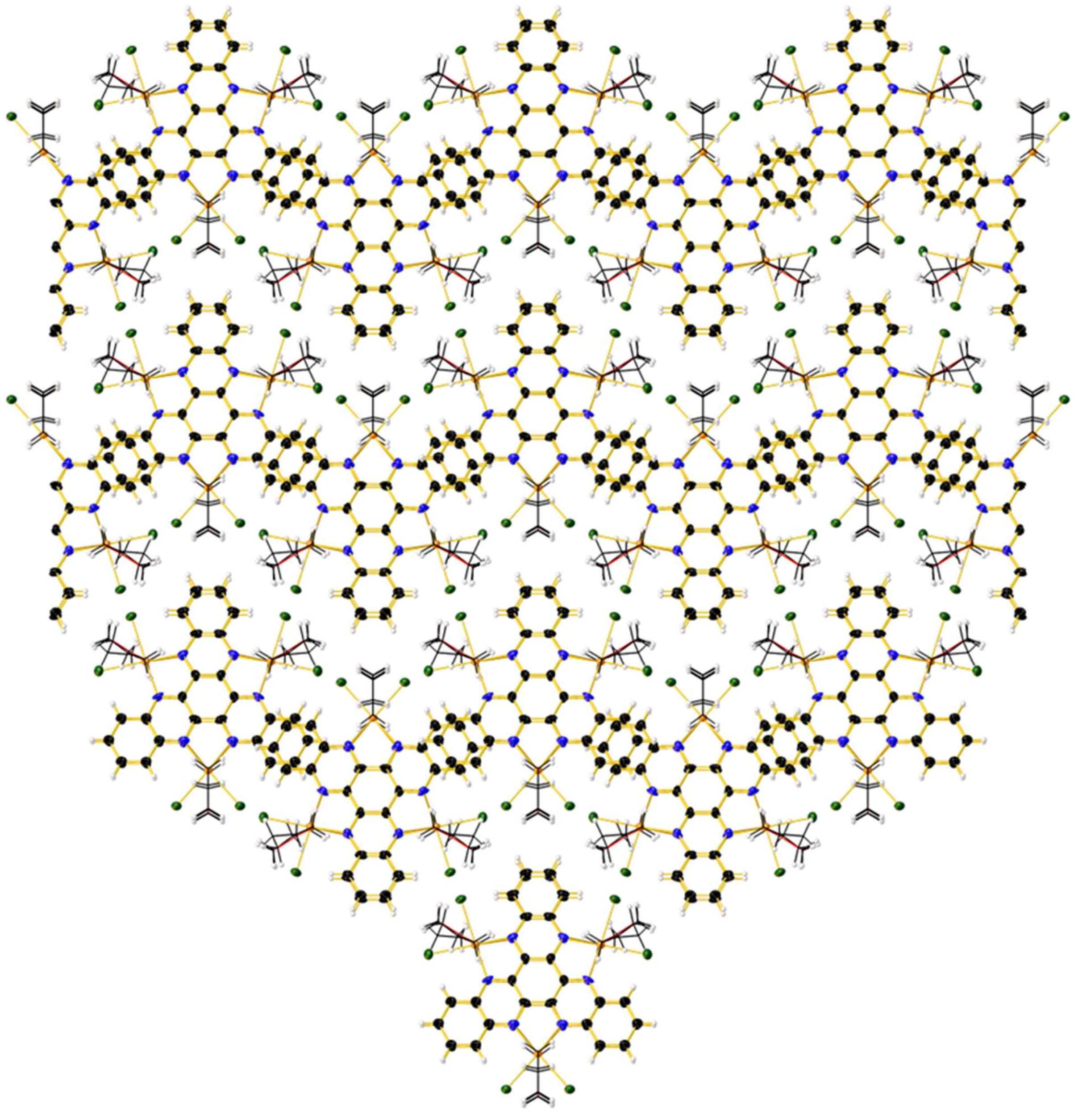

Figure S6. Packing in the crystal lattice of **1-U** viewed along the crystallographic *a*-axis. Thermal ellipsoids at 30% probability.

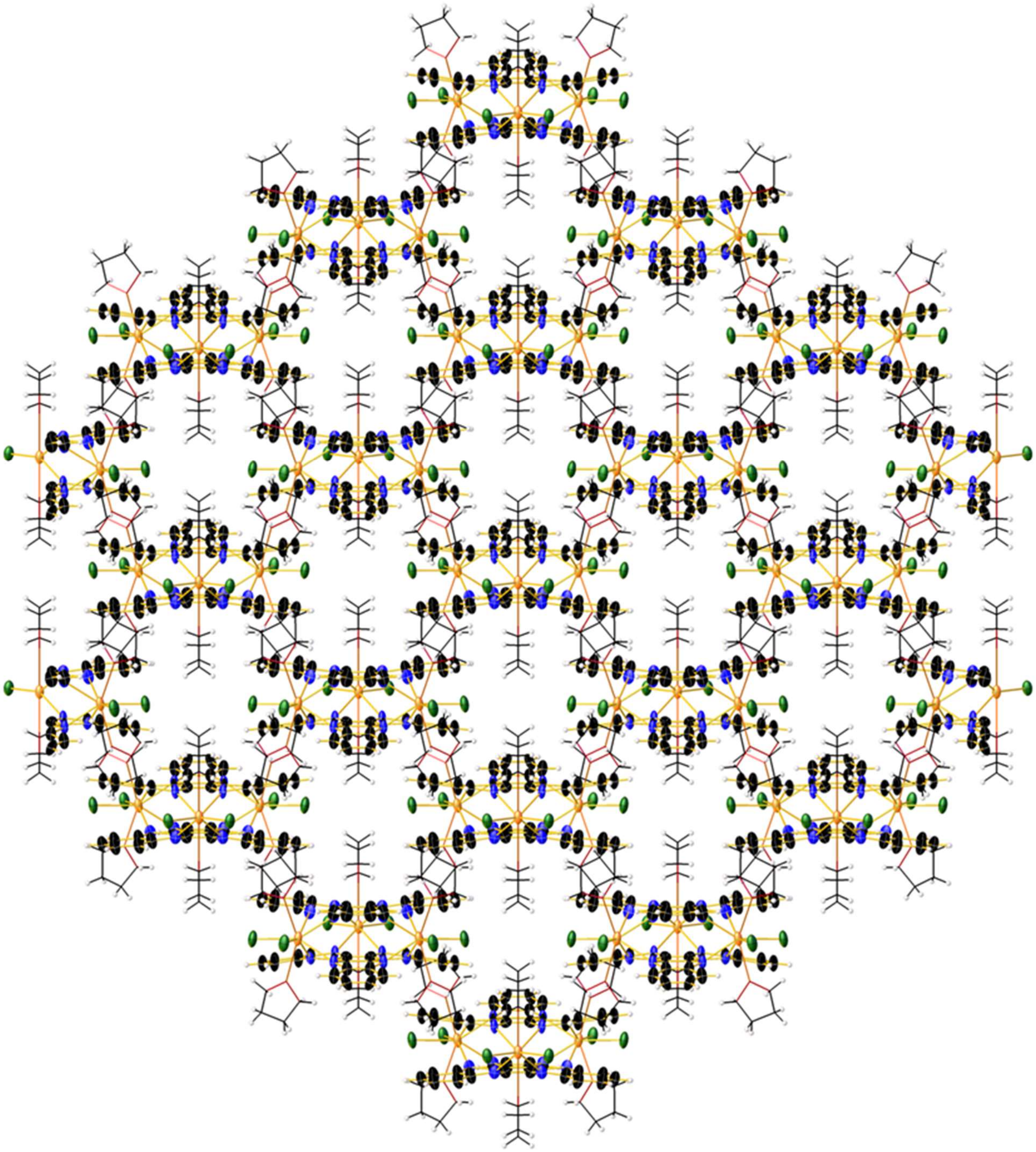

Figure S7. Packing in the crystal lattice of **1-U** viewed along the crystallographic *c*-axis. Thermal ellipsoids at 30% probability.

5. NMR spectroscopy

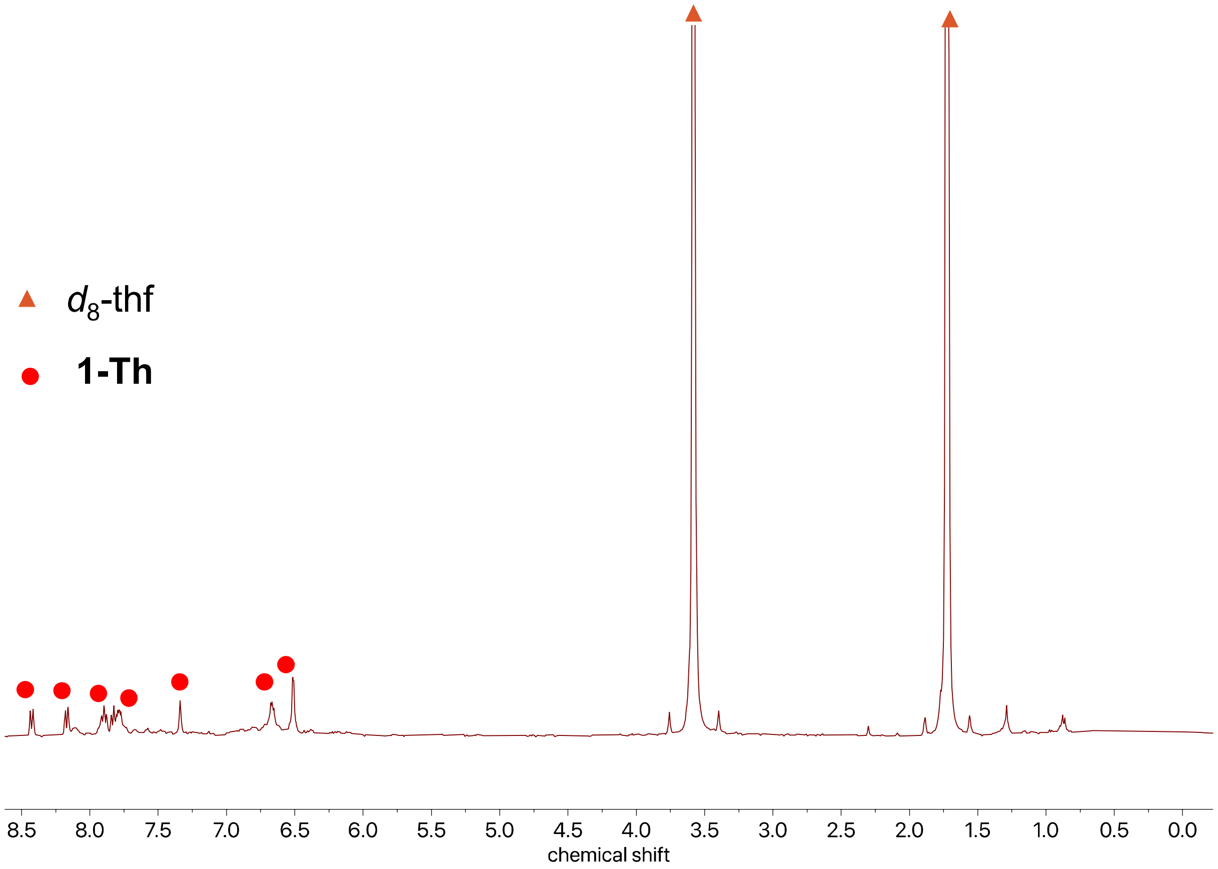

Figure S8. ^{1}H NMR spectrum of the 3:1:3 stoichiometry reaction of KC_8:HAN:Th in THF-D_8 after 17 h.

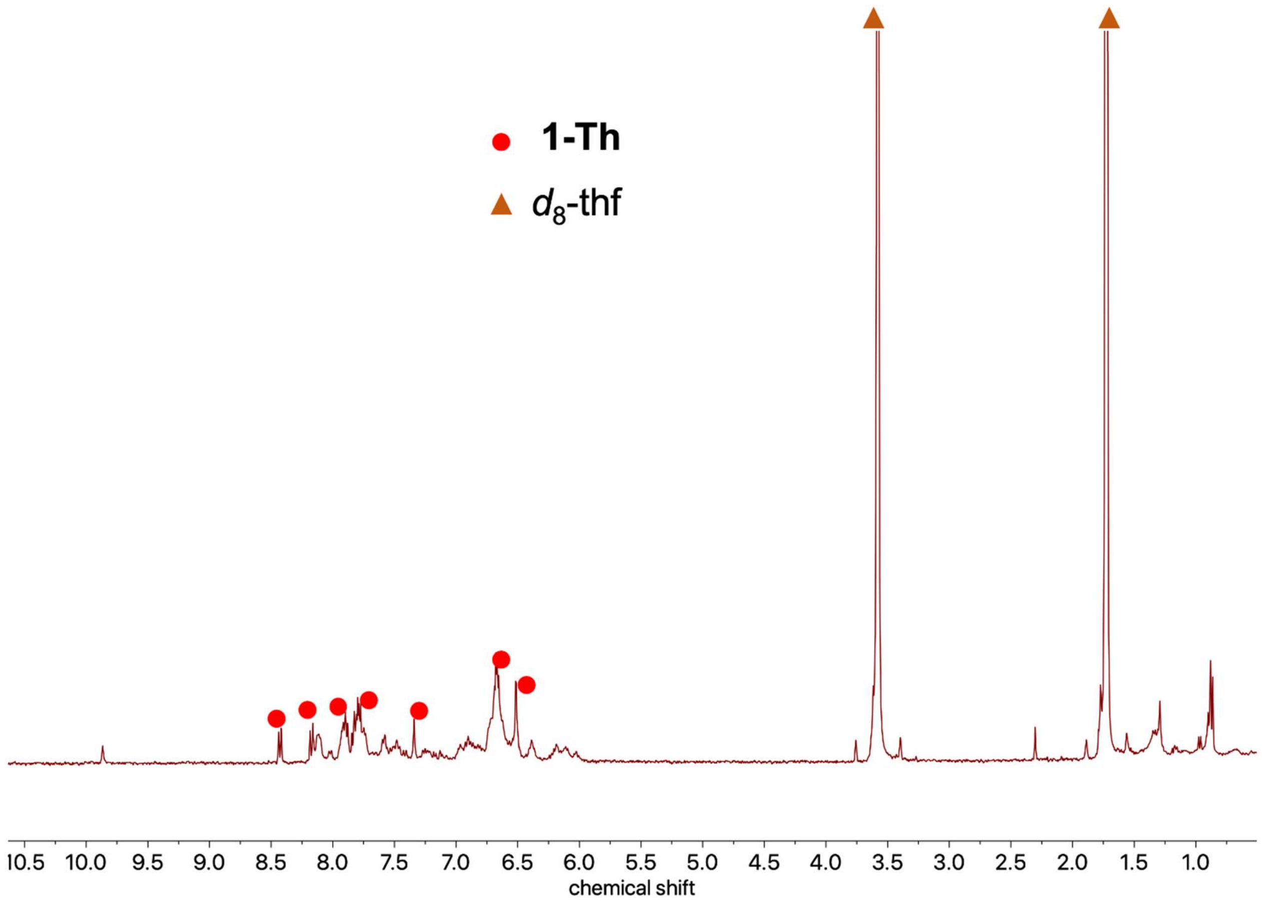

Figure S9. ^{1}H NMR spectrum of the 6:2:3 stoichiometry reaction of KC_8:HAN:Th in THF-D_8 after 17 h.

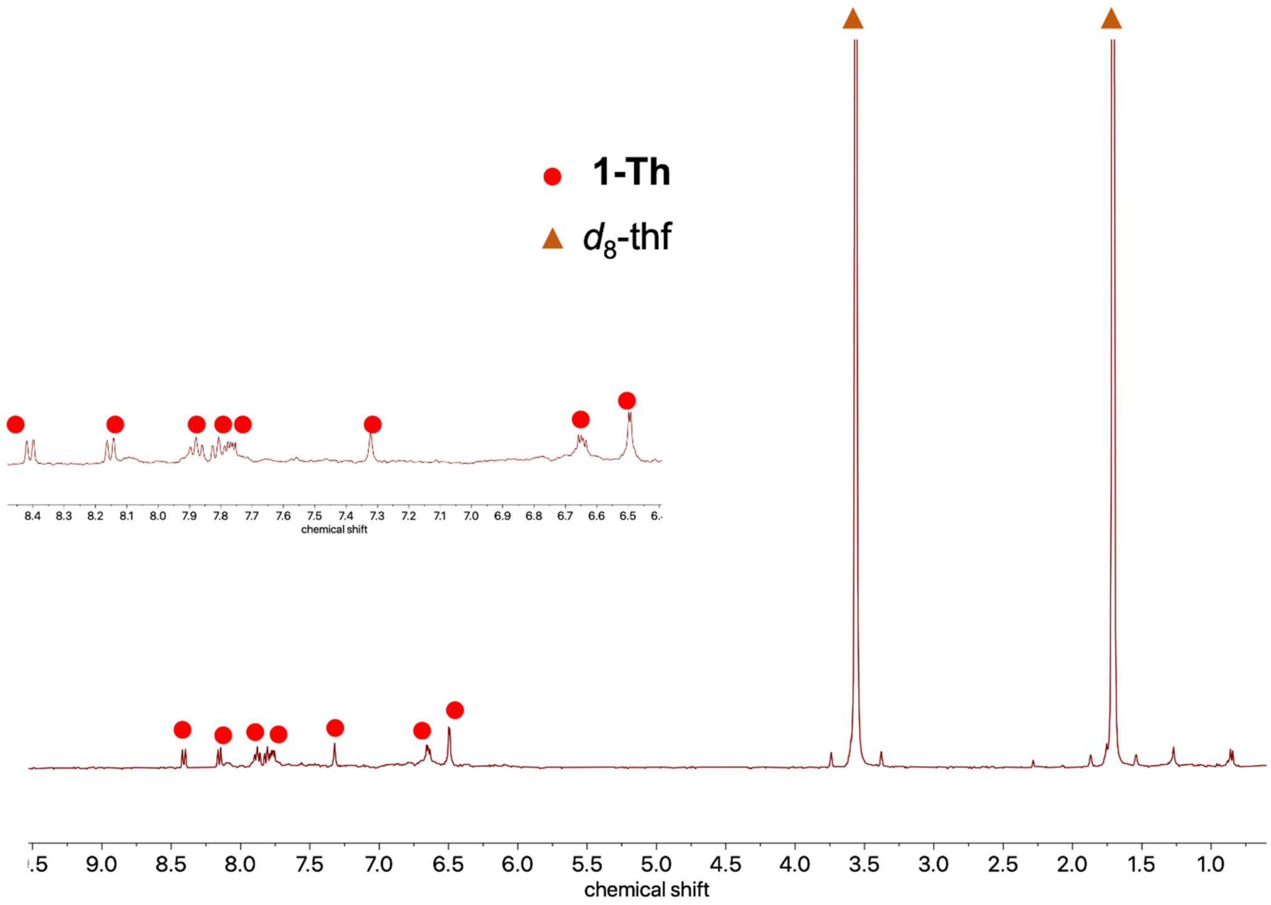

Figure S10. ^{1}H NMR spectrum of isolated **1-Th** in THF-D_8.

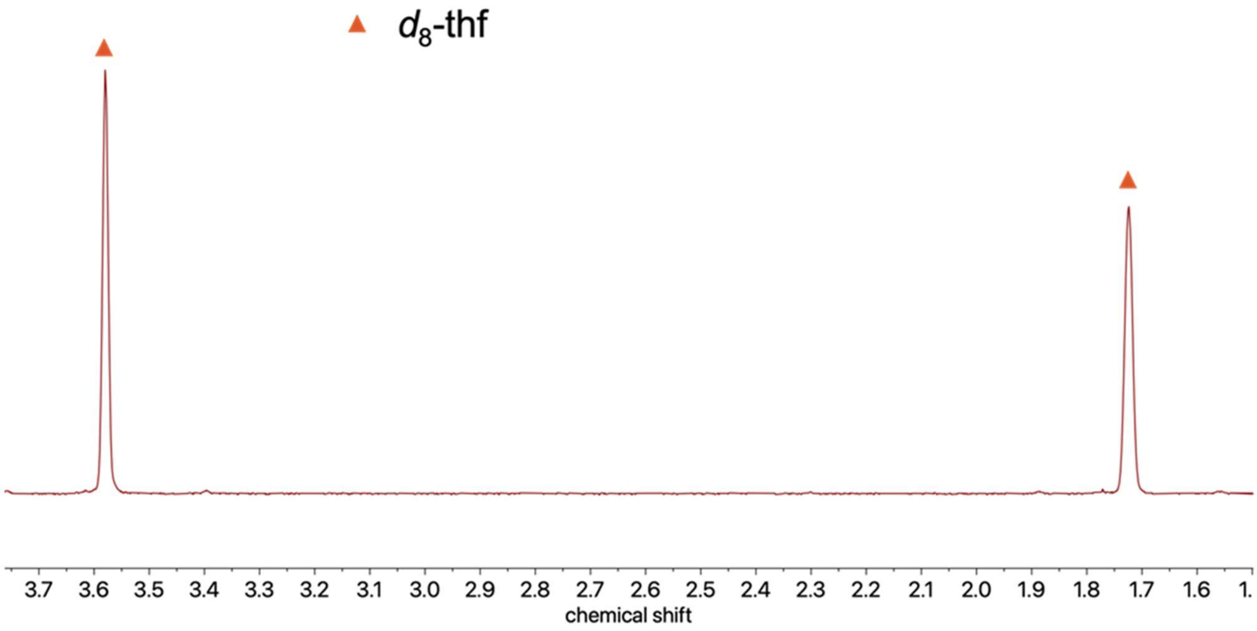

Figure S11. ^{1}H NMR spectrum of isolated **1-Th** in THF-D_8 with an inserted capillary of neat THF-D_8. Superimposition of the resonances confirms the diamagnetism of **1-Th** in solution.

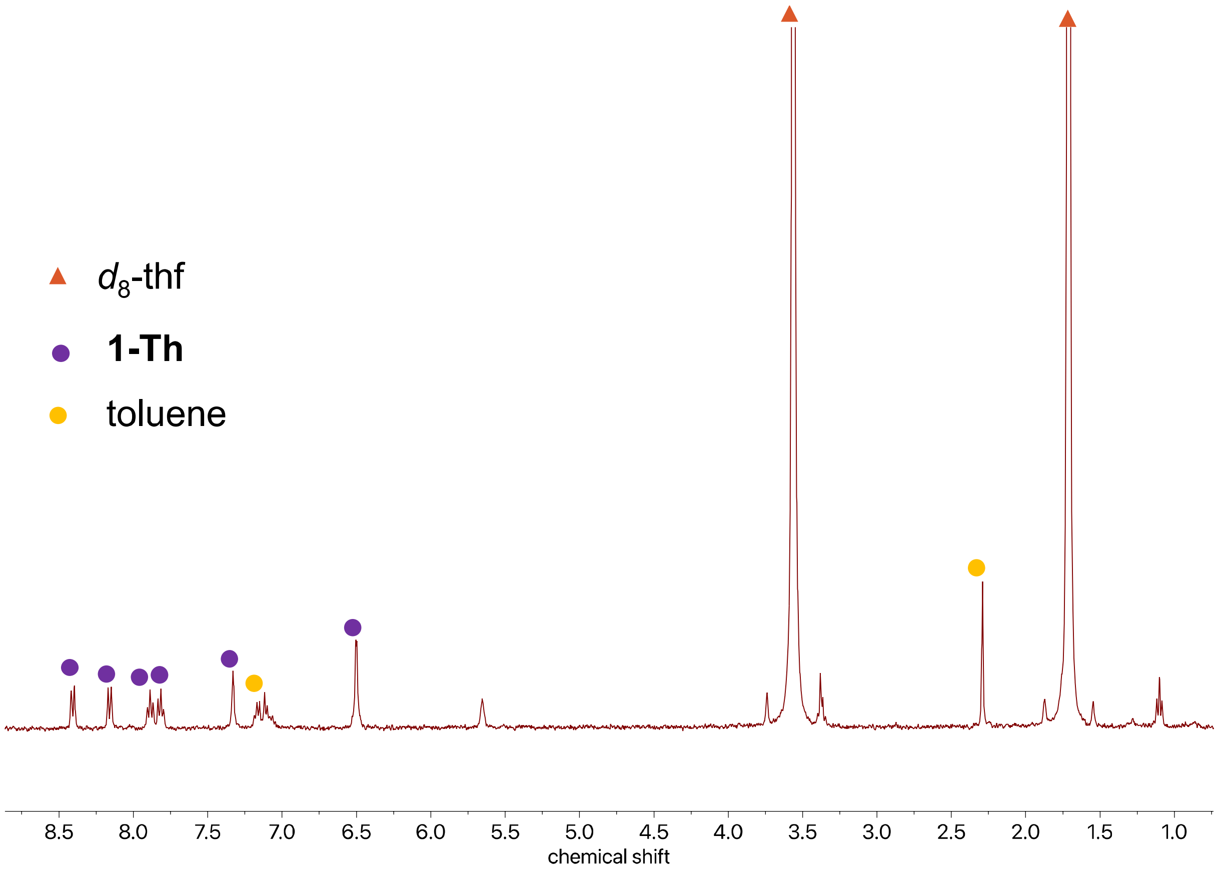

Figure S12. ^{1}H NMR spectrum in THF-D$_8$ of the blue supernatant containing a mixture of **1-U** and an unidentified side product.

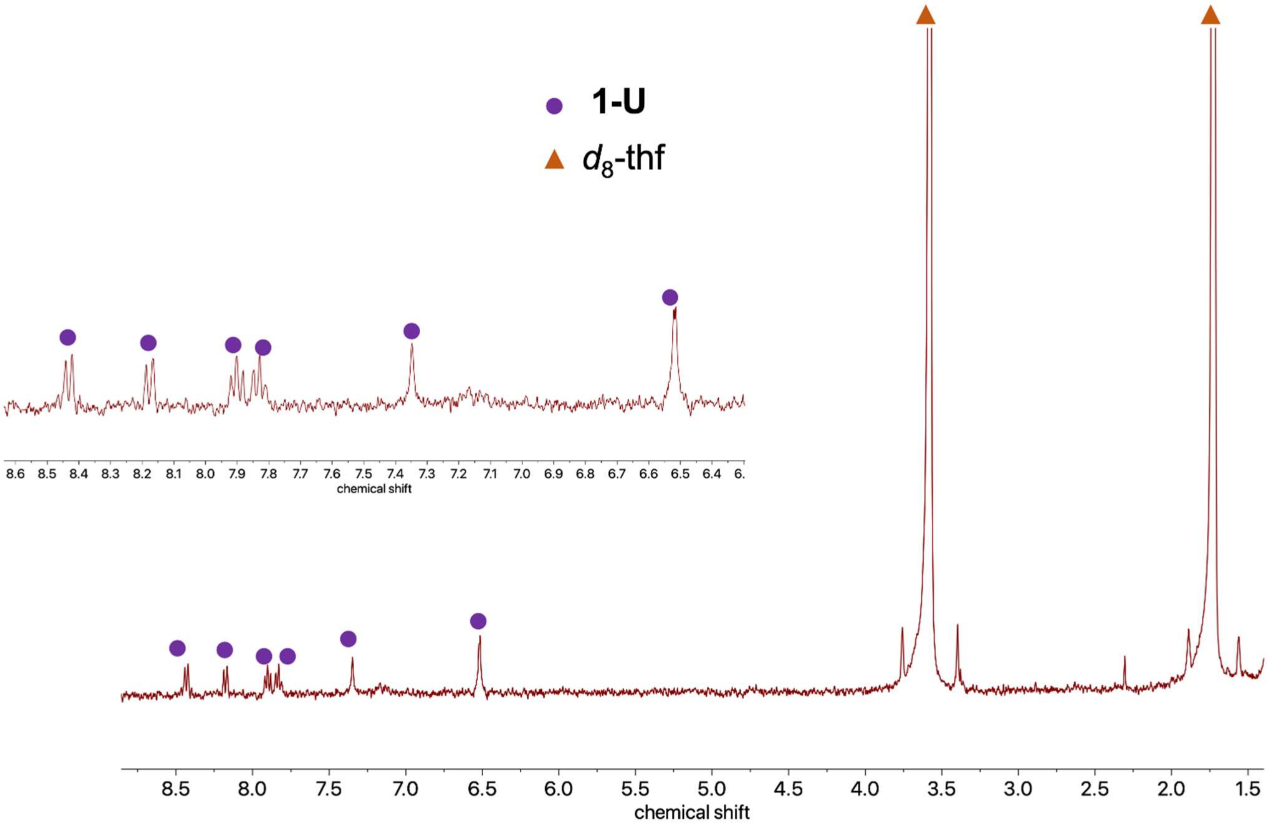

Figure S13. ^{1}H NMR spectrum of isolated **1-U** in THF-D_8.

Computational details

The geometries of **1-Th** and **1-U** used in the calculations were extracted from the crystal structures. The positions of hydrogen atoms were optimized using density functional theory (DFT). In case of **1-Th**, also the positions of the carbon atoms in the THF ligands were optimized due to the significant disorder in the crystal structure. The positions of remaining atoms were kept frozen to their crystal-structure coordinates. The geometry optimization was carried out using the *Gaussian* 16 programs suite revision C.02[11]. The range-separated hybrid CAM-B3LYP exchange-correlation (XC) functional[12–14] was used along with Ahlrichs' valence-polarized double-ζ quality SVP basis sets[15]. The core electrons of the thorium(IV) and uranium(IV) ions were treated with Stuttgart-type small-core effective core potentials (ECPs) [16,17] along with the SVP valence basis sets[18].

The orbital structure and exchange coupling in **1-Th** and **1-U** was analyzed based on single-point DFT calculations carried out using the geometries with optimized hydrogen positions (and THF ligands). Additional single-point calculations were carried out on the $[HAN]^{3-}$ anions in various spin states. Geometries were extracted from the structure of **1-Th** without further optimization. The CAM-B3LYP XC functional was utilized along with Ahlrichs' valence-double-polarized triple-ζ quality TZVPP basis sets [18,19] for non-hydrogen atoms, SVP [15] for hydrogens and the Stuttgart small-core ECP for thorium(IV) and uranium(IV) ions [16,17]. The calculations were carried out using *Gaussian* 16.

The $M_S = 3$, $M_S = 1$, and $M_S = 0$ broken-symmetry states were calculated for **1-Th**. The energies are listed in Table S4. Stability analyses [20,21] were carried out to ensure that all optimized states correspond to minima in the molecular orbital coefficient space. The exchange coupling constants were extracted from the results using the broken-symmetry (BS) DFT approach[22–26] with the Yamaguchi projection[27–29], where LS refers to the lower-spin state and HS to the higher-spin state.

$$J = \frac{2(E_{\mathrm{LS}} - E_{\mathrm{HS}})}{\langle S^2 \rangle_{\mathrm{HS}} - \langle S^2 \rangle_{\mathrm{LS}}}$$

Table S4. Energies and spin expectation values $\langle S^2 \rangle$ of the different broken-symmetry states calculated for **1-Th**.

State	Energy / a.u.	Relative energy / cm^{-1}	$\langle S^2 \rangle$
$M_S = 3$	–7879.74385804	15567.94	12.0811
$M_S = 1$	–7879.78682129	0.00	0.9723
$M_S = 0$	–7879.81479081	6138.60	2.1155

Further bonding analyses were carried out on **1-Th** at the DFT level using the *ADF* module version 2022.101 of the *AMS* program suite [30,31] and the geometry with optimized hydrogen positions and coordinates of the THF ligands. To use symmetry to provide further insight into the electronic structure, the geometry was symmetrized. The molecular point group in the crystal structure is C_3. This was increased to C_{3h} which required minimal modification of the geometry. As a result, the $[HAN_2]^{6-}$ dimer has exact D_{3h} symmetry. However, the symmetry is broken by the different spins located on the two $[HAN]^{3-}$ fragments and in practice reduced to C_{3v}. Test calculations were carried out to ensure that the minimal changes in the geometry do not cause any significant deviation in the results.

The bonding analysis was based on the Morokuma-Ziegler-Rauk energy decomposition analysis[32–34] as implemented in ADF. The analysis was carried out on top of a single-point energy evaluation using the hybrid PBE0 XC functional[12–14]. Dispersion effects were approximated using the empirical DFT-D3

correction with the Becke–Johson (BJ) damping function [35,36]. Scalar relativistic effects were introduced using the zeroth-order regular approximation (ZORA) as implemented in ADF [37–39]. The standard ADF Slater-type basis sets designed for ZORA calculations were utilized in all ADF calculations [40]. Each atom was treated with a triple-ζ quality basis with two sets of polarization functions (TZ2P) and a small frozen core. In the thorium(IV) ions, the frozen core consisted of the all shells up to 4f, and in case of C, N, O and Cl atoms the frozen core consisted of the 1s shell only. The ADF "NumericalQuality" keyword was set to "Good" and the true density as opposed to the fitted density ("ExactDensity" keyword) was used in the construction of the XC potential.

In the energy decomposition analysis (EDA), the energy associated with formation of the molecule from the fragments is referred to as the instantaneous interaction energy, ΔE_{inst}. It is related to the bonding energy between the fragments but does not include the energy required to distort the fragments from the optimal geometries to those they possess in the final molecule. The ΔE_{inst} can be partitioned into electrostatic interaction ΔE_{elstat}, orbital interaction ΔE_{orb} and Pauli repulsion ΔE_{Pauli} terms. The ΔE_{elstat} describes the classic electrostatic interaction between the molecular fragments before the electron densities mix, ΔE_{orb} describes the energy lowering once the fragment densities mix, and ΔE_{Pauli} describes non-classical repulsion between the fragment densities due to the antisymmetry of the wave function. In addition, the DFT-D3 dispersion correction ΔE_{disp} can be separated from the other components. The orbital interaction energy can be further partitioned into contributions from different irreducible representations of the molecular point group. Symmetry was only utilized in the study of the bonding between the $[HAN]^{3-}$ fragments and, due to the broken-spin nature of the fragments, the highest point-group symmetry is C_{3v}.

Table S5. Energy Decomposition Analysis of 1-Th. Step 1 describes the interaction between two $[HAN]^{3-}$ quartet radicals to give $[(HAN)_2]^{6-}$. Step 2 describes reconstruction of **1-Th** through the interaction of $[(HAN)_2]^{6-}$ with three $[ThCl_2(THF)_2]^{2+}$ cations. Energies are stated in kJ mol^{-1}.

**Bonding contribution**		**Step 1**	**Step 2**
ΔE_{inst}		1830.83	–11368.57
ΔE_{elstat}		1913.76	–10472.53
ΔE_{orb}	Total	–376.87	–3977.91
	A_1	–89.24	
	A_2	–18.69	
	E	–268.12	
ΔE_{Pauli}		431.68	3385.08
ΔE_{disp}		–137.74	–303.19

Optical excitations in **1-Th** were modeled using time-dependent DFT (TD-DFT) utilizing the ADF code[41]. The calculations were carried out with the range-separated CAMY-B3LYP XC functional[13,14,42,43] (which has a different separation function than in CAM-B3LYP) using the same geometry as in the other single-point calculations. Other computational details were the same as in the bonding analyses. The calculation was carried out using restricted formalism and the thirty lowest singlet-singlet excitations were solved. It should be noted that both the geometry and restricted formalism introduce some approximation when comparing the spectrum with experimental observation as the UV/vis/NIR spectrum

was measured in solution phase, and the system has a low level of spin contamination in its ground state. Solvent effects were considered with the conductor-like screening model of solvation (COSMO)[44–46] with THF as the solvent using the default parameters in ADF.

Table S6. Time-dependent DFT results for 1-Th. Optical excitation energies and orbital descriptions of the transitions with significant oscillator strengths.

Transition	E / eV	λ / nm	Oscillator strength	Initial orbitals	Final orbitals
1	1.2316	1006.69	0.0000		
2	1.2325	1006.00	0.0000		
3	1.5539	797.89	0.0000		
4	1.6830	736.67	0.0000		
5	1.6831	736.63	0.0000		
6	2.1128	586.82	0.0526		
7	2.5914	478.44	0.0000		
8	2.5975	477.32	0.0000		
9	2.5981	477.21	0.0000		
10	2.6428	469.14	2.0560	Pancake-bonding orbitals	Pancake-anti-bonding orbitals
11	2.6809	462.48	0.0001		
12	2.7293	454.27	0.0617		
13	2.7300	454.15	0.0617		
14	2.9750	416.76	0.5262	HAN π orbitals	Pancake-σ-anti-bonding orbital
15	2.9756	416.67	0.5278	HAN π orbitals	Pancake-σ-anti-bonding orbital
16	3.2178	385.30	0.0000		
17	3.4765	356.64	0.3130	Pancake-bonding orbitals	HAN π orbitals, Th 6*d*
18	3.4773	356.55	0.3123	Pancake-bonding orbitals	HAN π orbitals, Th 6*d*
19	3.4808	356.20	0.0012		
20	3.5320	351.03	0.4379	HAN π orbitals	Pancake-anti-bonding orbitals
21	3.5324	350.99	0.4379	HAN π orbitals	Pancake-anti-bonding orbitals
22	3.6507	339.62	0.0713		
23	3.6518	339.52	0.0710		
24	3.6639	338.39	0.0001		
25	3.7581	329.91	0.0000		
26	3.8237	324.25	0.6254	Pancake-bonding orbitals	HAN π orbitals, Th 6*d*
27	3.8242	324.21	0.6226	Pancake-bonding orbitals	HAN π orbitals, Th 6*d*
28	3.8738	320.06	0.6385	HAN π orbitals	Pancake-anti-bonding orbitals
29	3.8745	320.00	0.6360	HAN π orbitals	Pancake-anti-bonding orbitals
30	3.9596	313.12	0.0016		

7. Solution-phase EPR spectroscopy

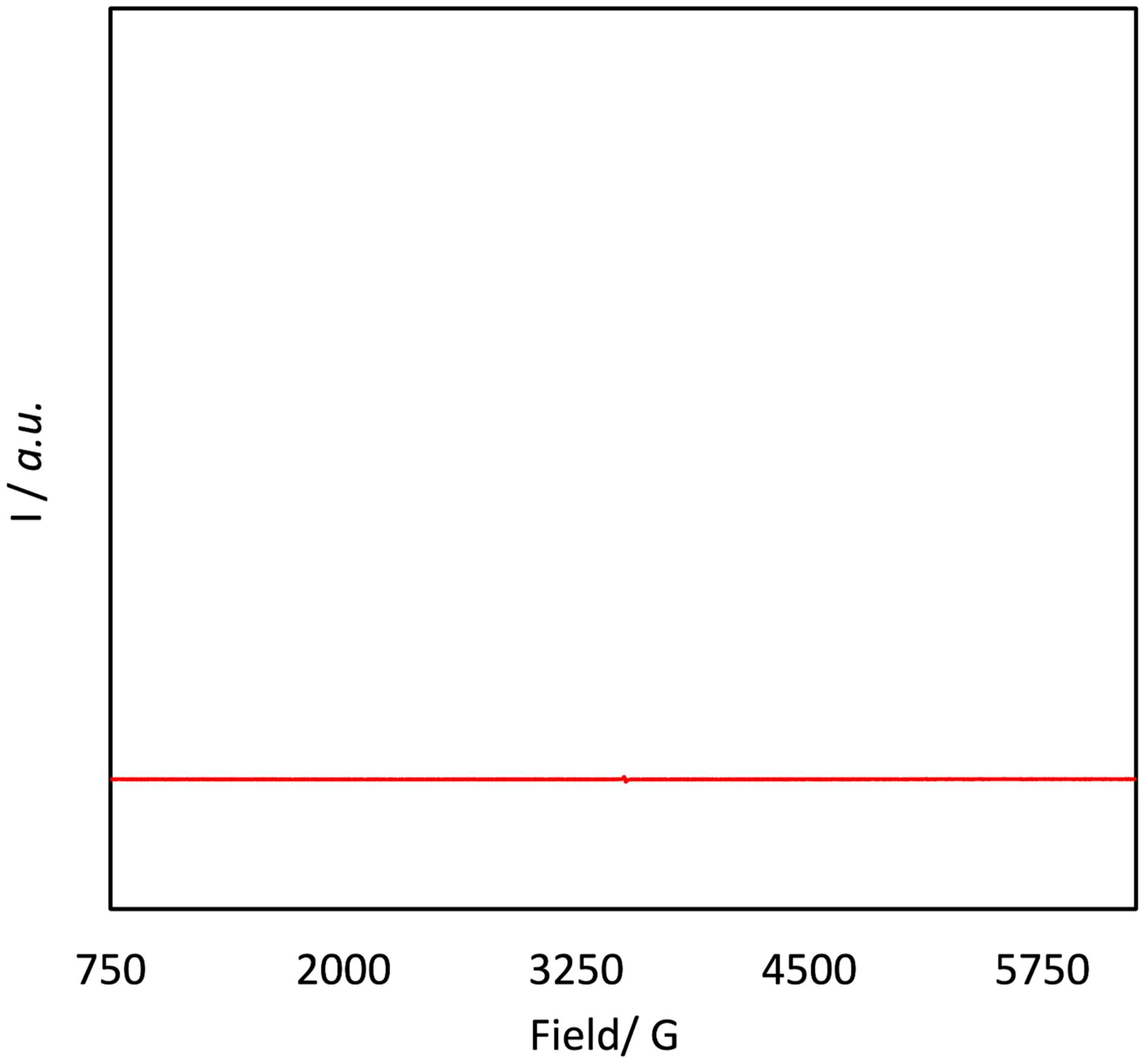

Figure S14. X-Band EPR spectrum of a 68 μM solution of **1-Th** in THF at 300 K. A miniscule signal is observed close to *g* = 2.00 and is most likely due to small amount of sample precipitating from the solution.

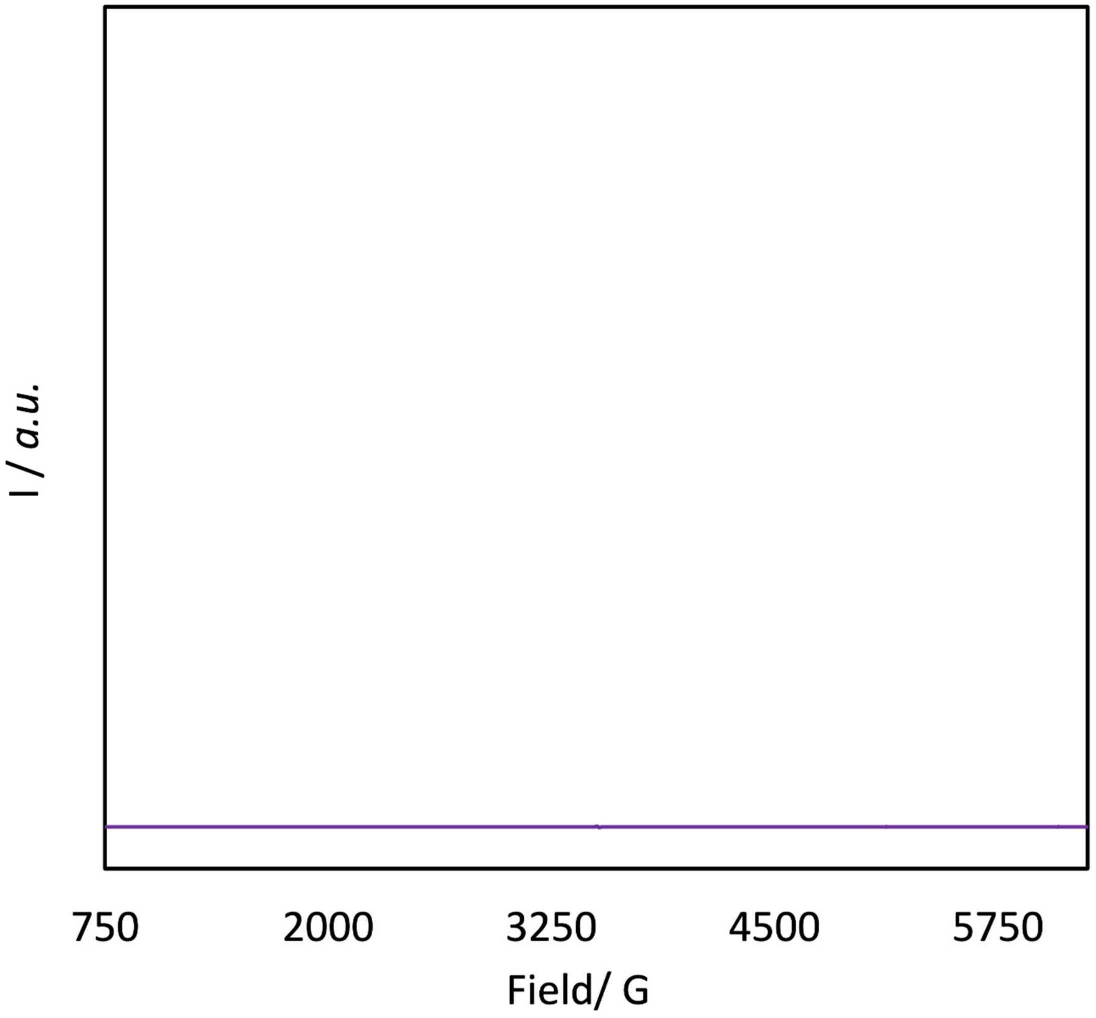

Figure S15. X-Band EPR spectrum of a 55 μM solution of **1-U** in THF at 300 K.

8. UV-vis-NIR spectroscopy

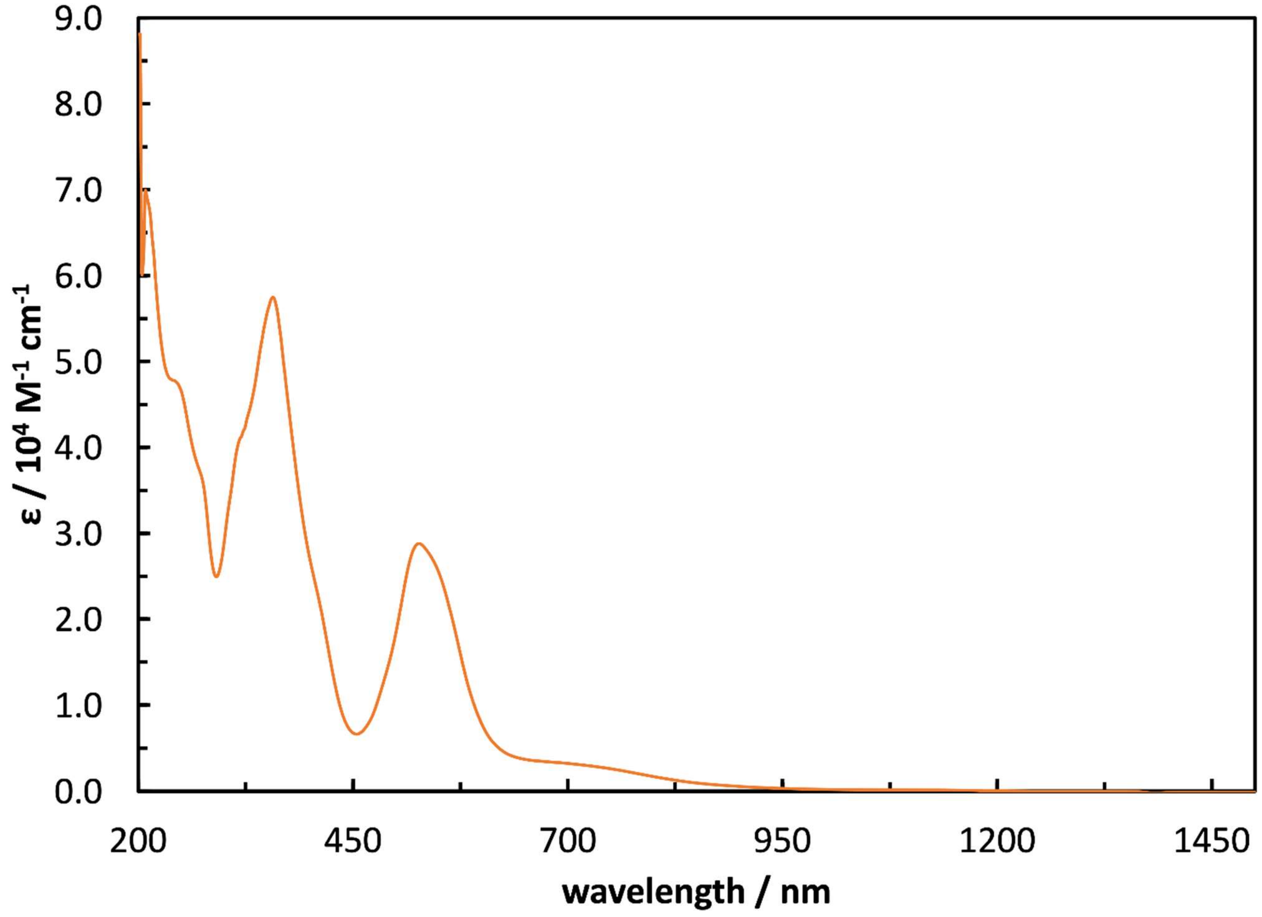

Figure S16. UV/vis/NIR spectrum of a 68 μM solution of isolated **1-Th** in THF.

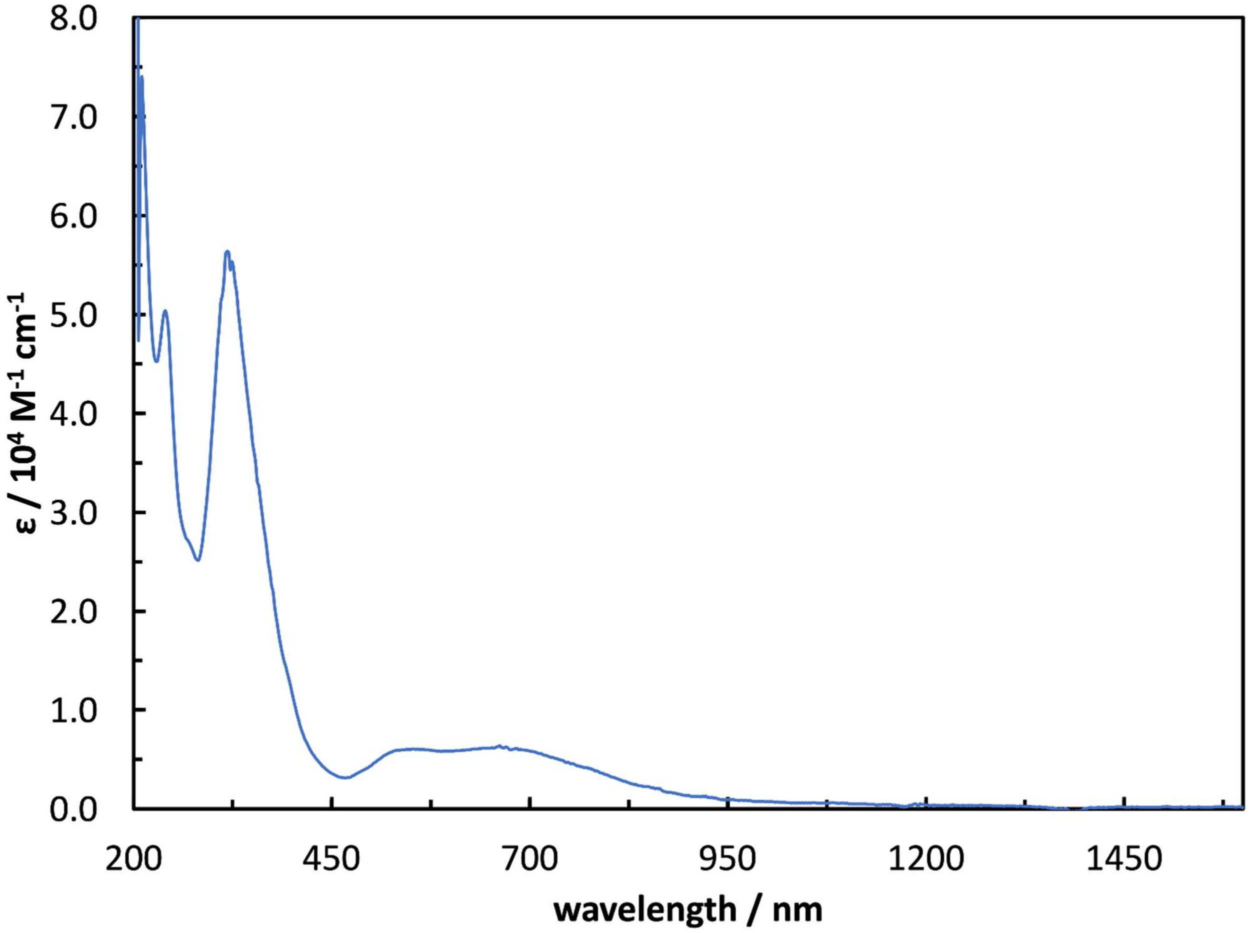

Figure S17. UV/vis/NIR spectrum of a 24 μM solution of isolated **1-U** in THF.

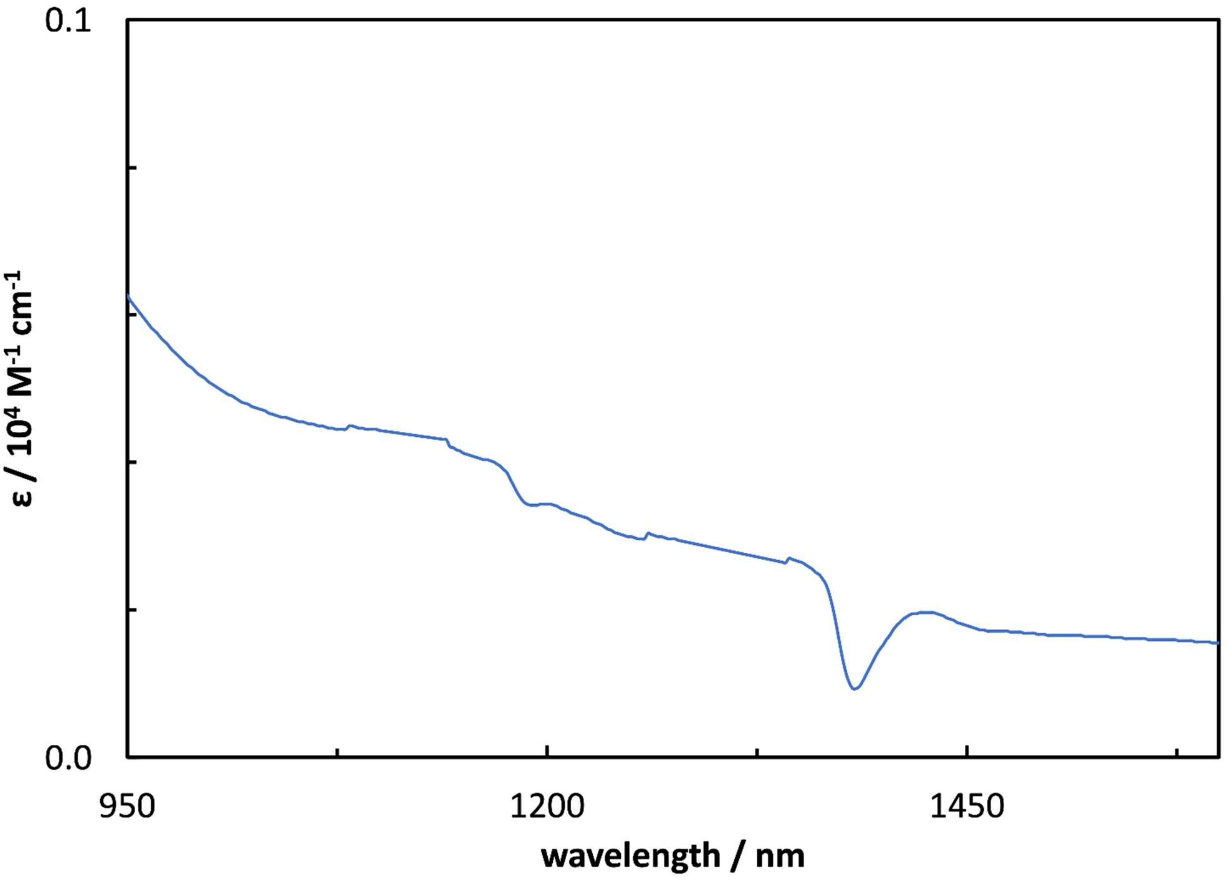

Figure S18. UV/vis/NIR spectrum of a 24 μM solution of isolated **1-U** in THF expanded in the NIR region.

9. Solid-state EPR spectroscopy

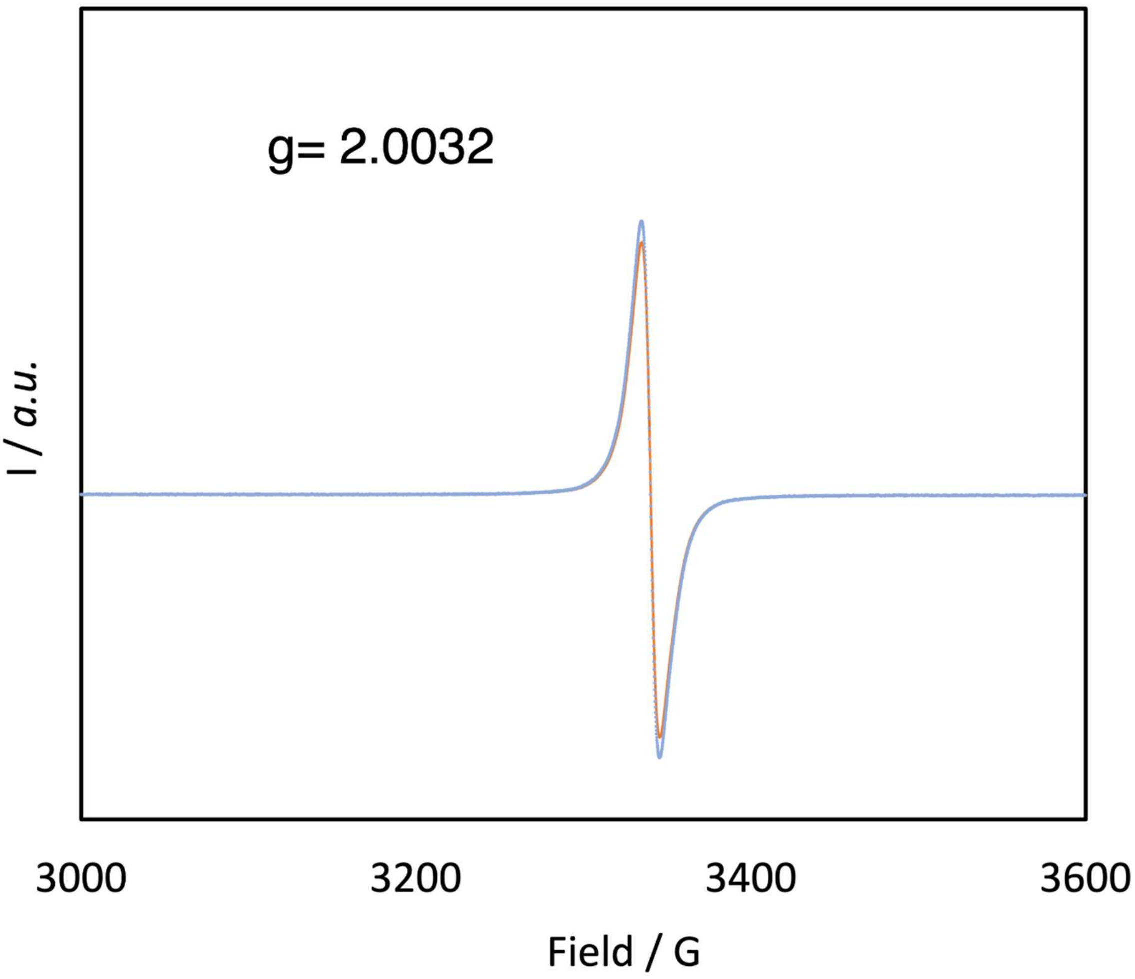

Figure S19. X-Band EPR spectrum of a powdered sample of **1-Th** measured at 300 K. The experimental data are shown in blue dot and the orange line is the simulation obtained with the EasySpin package for the MatLab program[47]. EasySpin simulation of the line shape uses the LWPP (line width peak-to-peak) where two different contributions can be modelled, *i.e.*, LWPP(1) with a Gaussian function and LWPP(2) with a Lorentzian function. The spectral line can be simulated exclusively with a Lorentzian lineshape with LWPP(2) = 1.14. No Gaussian contribution was observed (LWPP(1) = –0.03 when freely varying from 0 to 2). A *g*-factor of 2.0032 was obtained from both calculation and simulation.

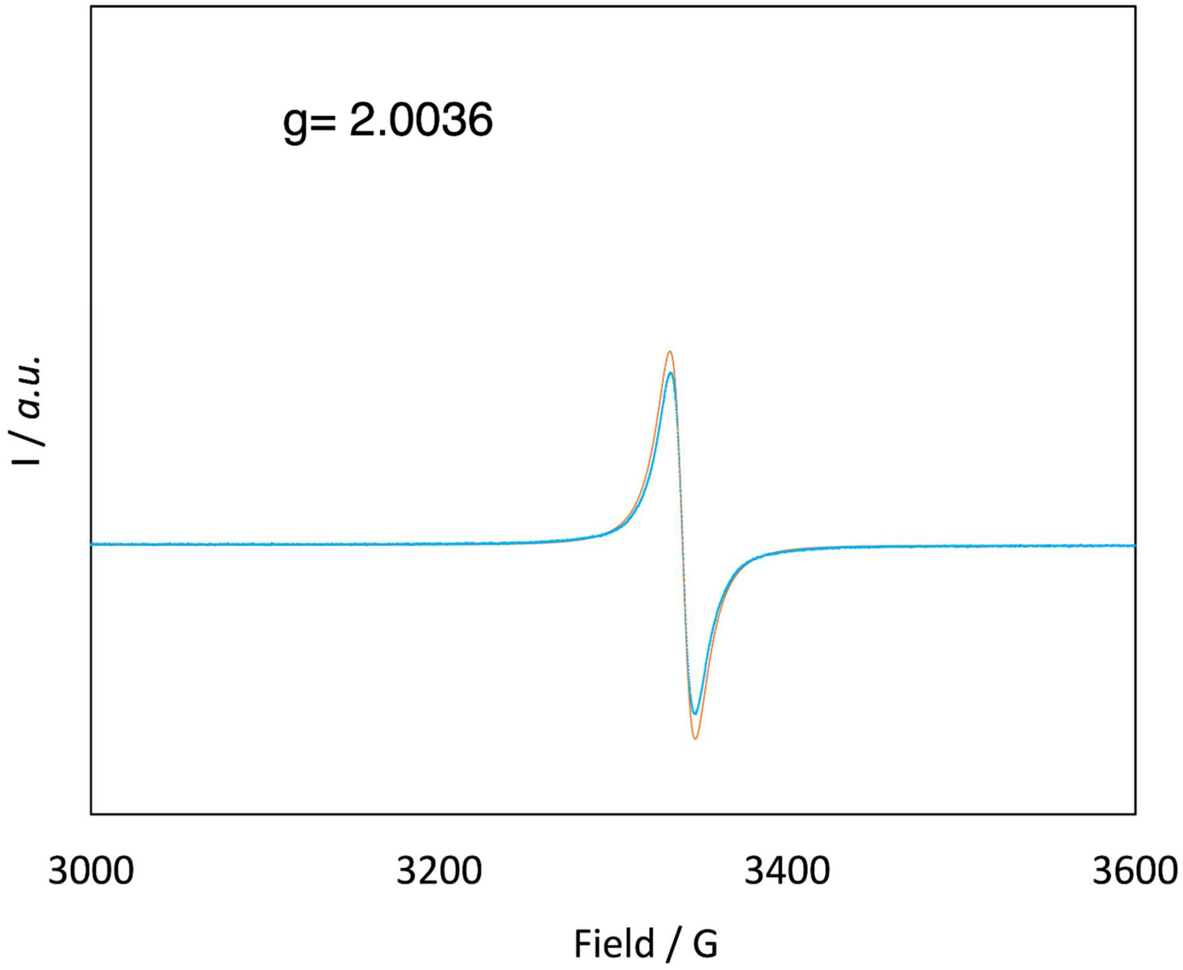

Figure S20. X-Band EPR spectrum of a powdered sample of **1-U** measured at 300 K. The experimental data are shown in blue dot and the orange line is the simulation obtained with the EasySpin package for the MatLab program[47]. The spectral line was simulated exclusively with a Lorentzian line shape with LWPP(2) = 1.45. No Gaussian contribution was observed (LWPP(1) = –0.04 when freely varying from 0 to 2). A *g*-factor of 2.0036 was obtained from both calculation and simulation.

EPR spin quantification for the solid-state X-band EPR spectra of 1-Th. Compound **1-Th** (3.8 mg, 1.8 µmol) was weighed in a quartz EPR tube, which was sealed under vacuum. The EPR spectrum of the sample was collected, and the number of spins associated to the sample was measured using the quantification software of the Bruker spectrometer. The quantification was measured every 10 K in a temperature range from 300 K to 80 K. The results ranged from 1.92×10^{16} to 2.59×10^{16} spins with an average of 2.12×10^{16} spins, corresponding to, when divided by Avogadro's constant, 0.035 µmol. Assuming one spin per molecule, this corresponds to 1.9% of the sample.

EPR spin quantification for the solid-state X-band EPR spectra of 1-U. Compound **1-U** (2.8 mg, 1.3 µmol) was weighed in a quartz EPR tube, which was sealed under vacuum. The EPR spectrum of the sample was collected, and the number of spins associated to the sample was measured using the quantification software of the Bruker spectrometer. The quantification was measured every 10 K in a temperature range from 300 K to 80 K. The results ranged from 1.25×10^{16} to 1.77×10^{16} spins with an average of 1.43×10^{16} spins, corresponding to, when divided by Avogadro's constant, 0.0234 µmol. Assuming one spin per molecule, this corresponds to 1.8% of the sample.

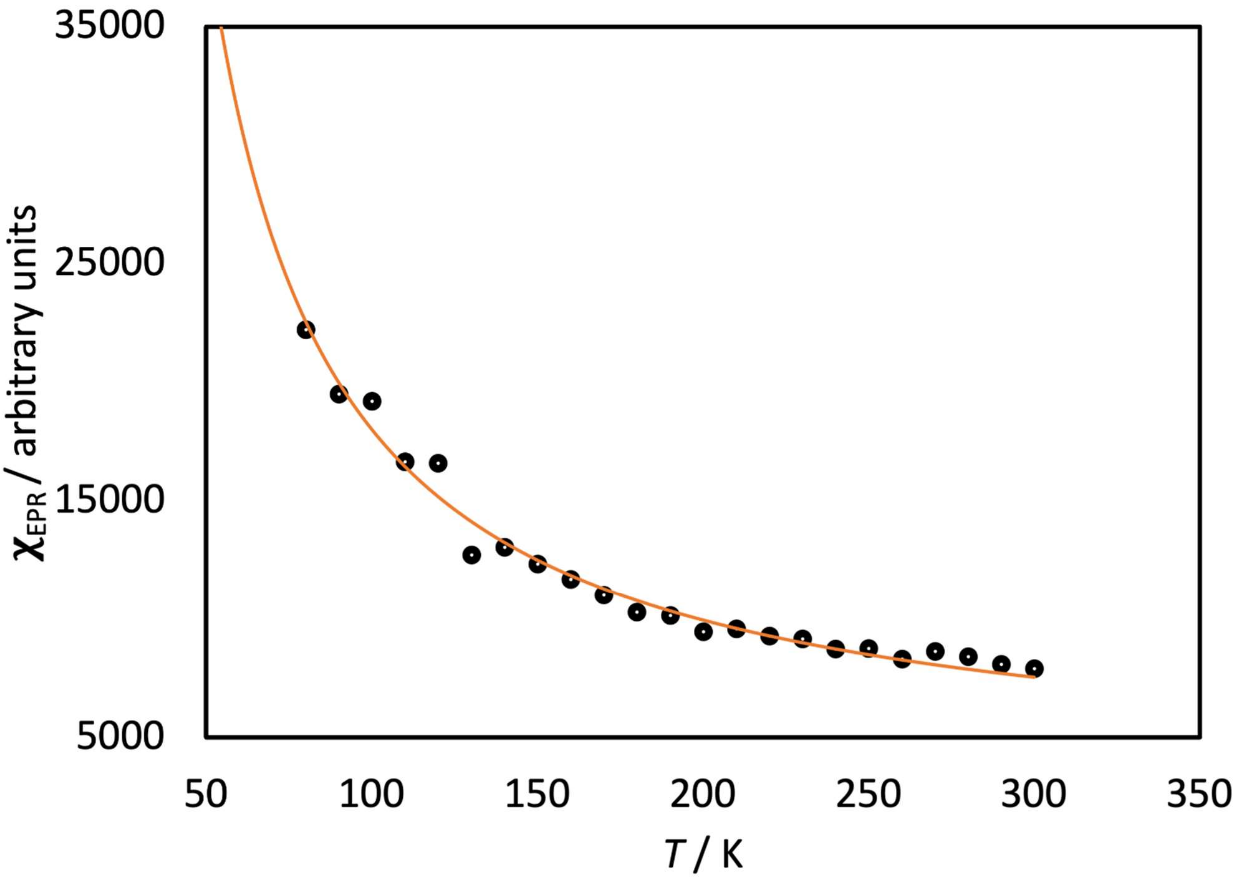

Figure S21. Variation of the spin susceptibility (χ_{EPR}) with temperature for **1-Th**, expressed as the normalized double integral of the area below the curve of the X-band EPR spectrum. The black circles represent the experimental data and the orange line is the best fitting curve obtained with the Curve Fitter package in MatLab. The fitting equation used in this case is $\chi_{EPR} = (A/T) + B$. The curve models a combination of Curie- and Pauli-type behaviour and has $R^2 = 0.9813$.

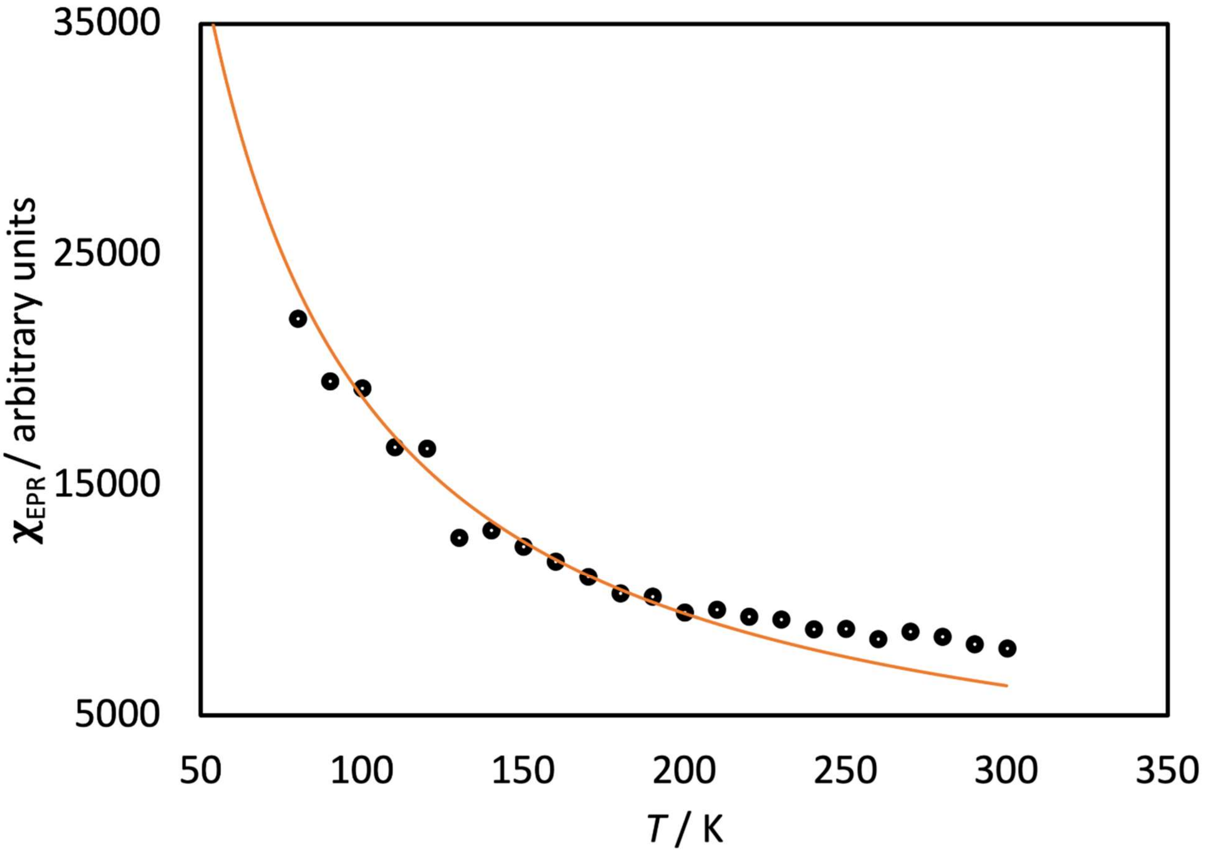

Figure S22. Variation of χ_{EPR} with temperature, expressed as the normalized double integral of the area below the curve of the X-band EPR spectrum, of **1-Th**. The black circles represent the experimental data and the orange line is the best fitting curve obtained with the Curve Fitter package in MatLab. The fitting equation used in this case is $\chi_{EPR} = (A/T)$. The curve uniquely models Curie-type behaviour. The value of $R^2 = 0.9457$ suggests that the data cannot be fitted satisfactorily using only a Curie model. Notably, the data above 200 K deviate from the model.

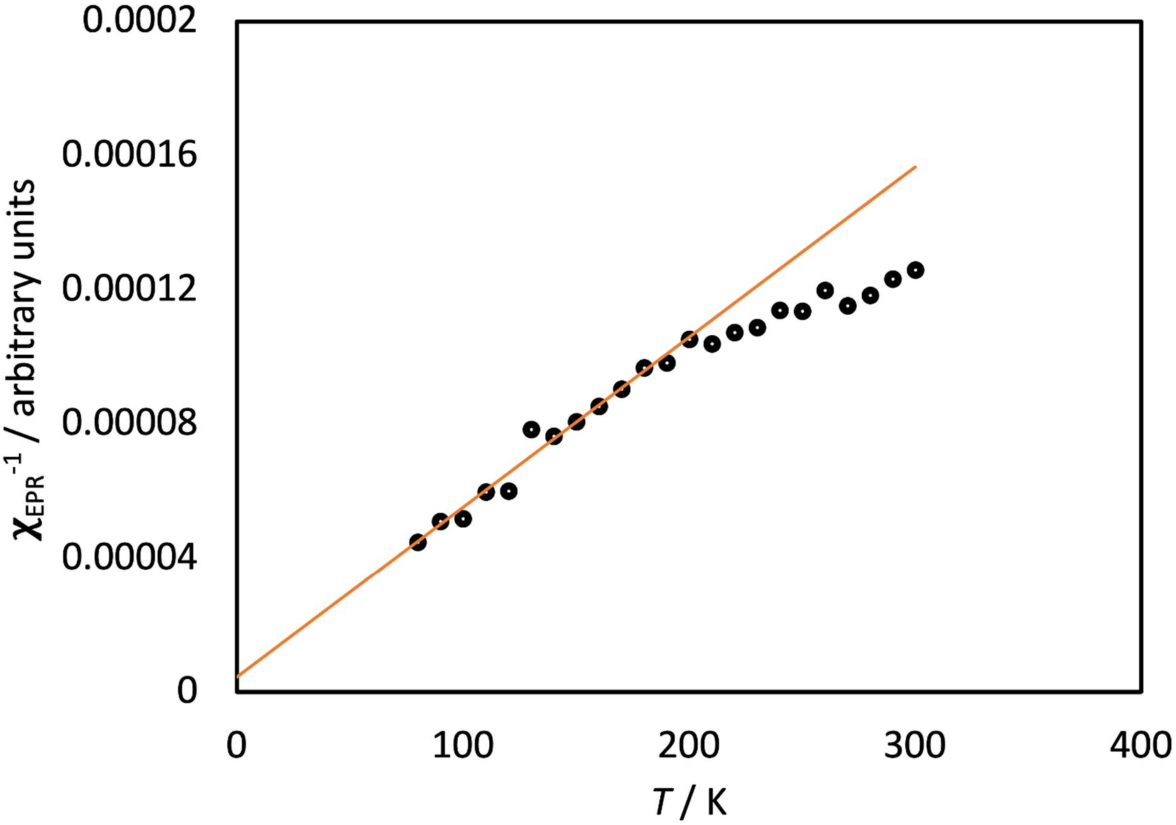

Figure S23. Variation of the inverse of χ_{EPR}, expressed as the normalized double integral of the area below the curve of the X-Band EPR spectrum, of **1-Th** with temperature in the range between 80 and 300 K. The black cirlcles represent the experimental data while the orange line is the best fitting curve obtained with the Curve Fitter package in MatLab. The fitting equation used in this case is $1/\chi_{EPR} = AT$. The fit models Curie behaviour and applies only to the data in the range 80-200 K. Data below 200 K can be modelled with a linear Curie behaviour (R^2= 0.9766) but the data between 200-300 K deviate from the model, suggesting that Pauli-like behaviour is needed to explain the data.

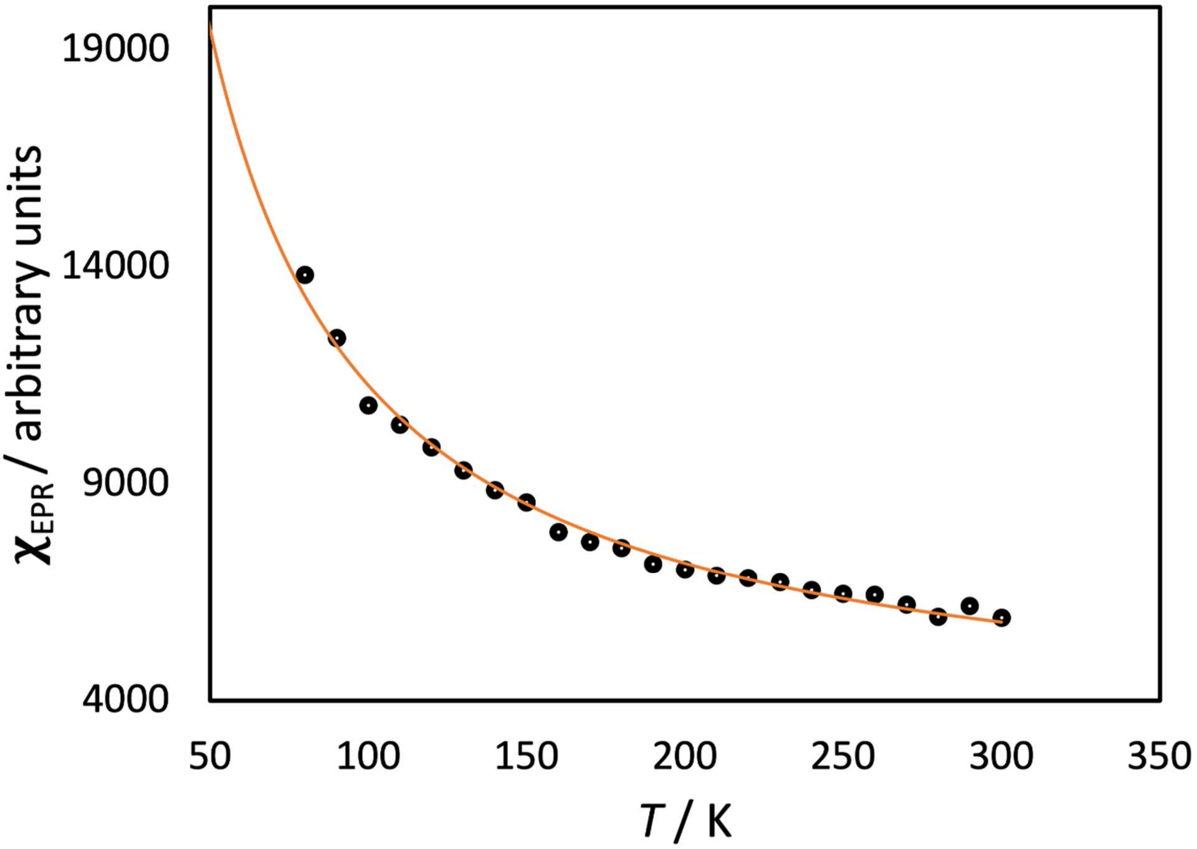

Figure S24. Variation of the χ_{EPR}, expressed as the normalized double integral of the area below the curve of the X-Band EPR spectrum, of **1-U** with temperature in the range between 80 and 300 K. The black cirlcles represent the experimental data while the orange line is the best fitting curve obtained with the Curve Fitter package in MatLab. The fitting equation used in this case is $\chi_{EPR} = (A/T) + B$. The curve models a combination of Curie- and Pauli-type behaviour and has $R^2 = 0.9906$, suggesting that both Curie and Pauli contribution should be considered.

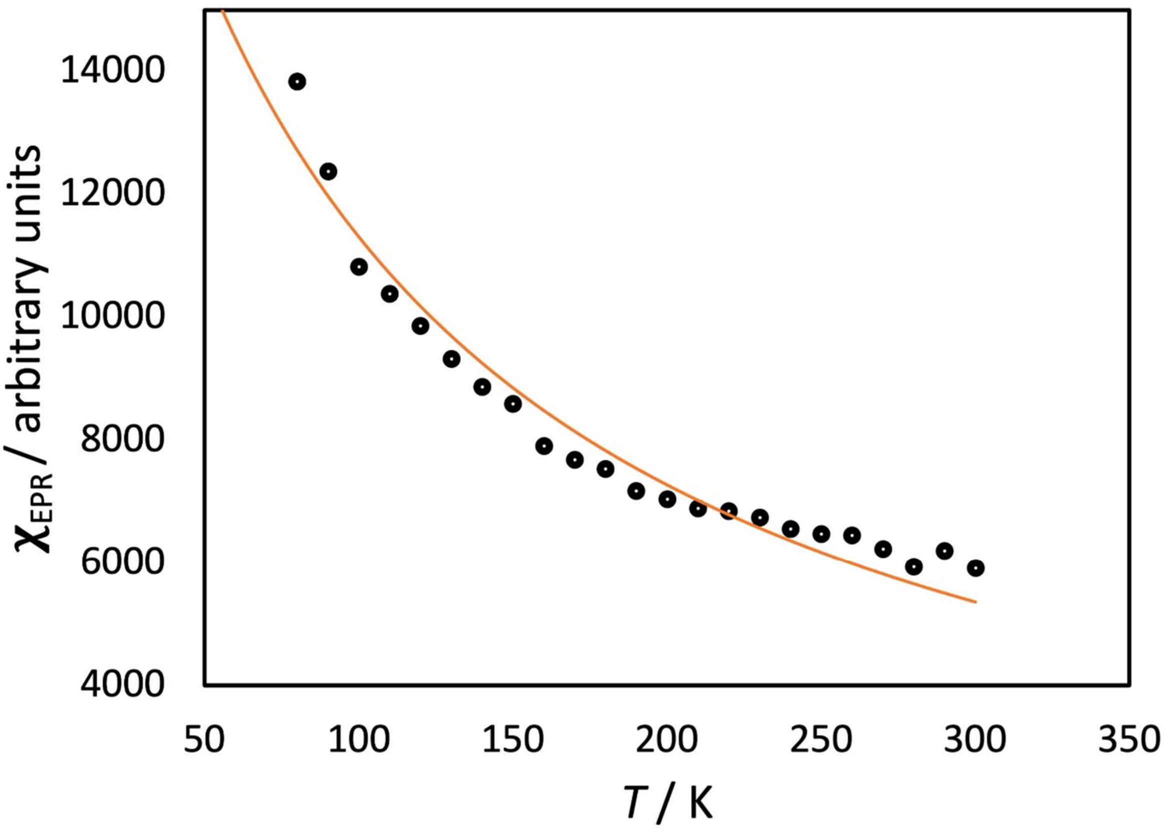

Figure S25. Variation of the χ_{EPR}, expressed as the normalized double integral of the area below the curve of the X-Band EPR spectrum, of **1-U** with temperature in the range between 80 and 300 K. The black cirlcles represent the experimental data while the orange line is the best fitting curve obtained with the Curve Fitter package in MatLab. The fitting equation used in this case is $\chi_{EPR} = (A/T)$. The curve uniquely models Curie-type behaviour. The value of $R^2 = 0.9557$ suggests that the data cannot be fitted satisfactorily using only a Curie model.

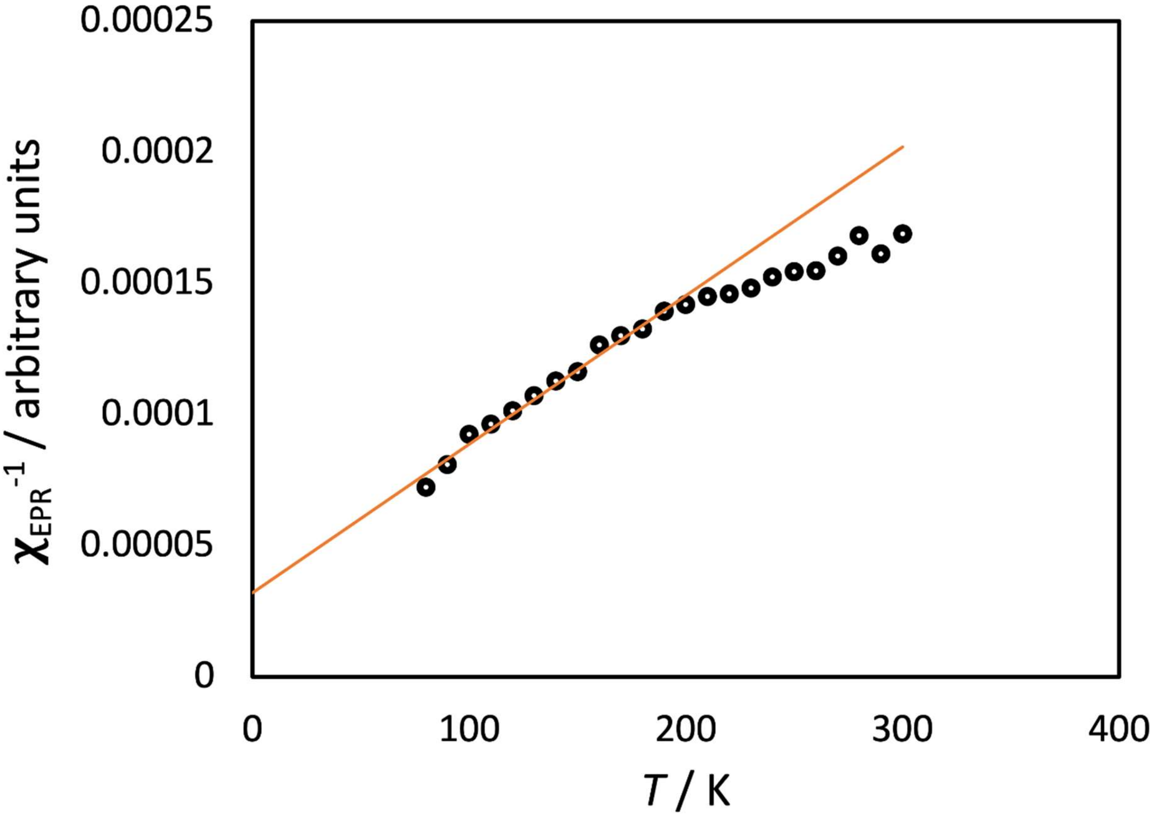

Figure S26. Variation of the inverse of χ_{EPR}, expressed as the normalized double integral of the area below the curve of the X-Band EPR spectrum, of **1-U** with temperature in the range between 80 and 300 K. The black cirlcles represent the experimental data while the orange line is the best fitting curve obtained with the Curve Fitter package in MatLab. The fitting equation used in this case is $1/\chi_{EPR} = AT$. The fit models Curie behaviour and applies only to the data in the range 80-200 K. Data below 200 K can be modelled with a linear Curie behaviour (R^2= 0.9856) but the data between 200-300 K deviate from the model, suggesting that Pauli-like behaviour is needed to explain the data.

10. Conductivity measurements

Current-voltage data for **1-Th** and **1-U** were recorded in an argon-filled glovebox. Measurements were performed on pre-patterned interdigitated gold electrodes on a microscopy slide glass substrate. Lead wires were attached by soldering directly onto the gold pads. The slides were stored in an oven at 80°C for at least three days before being transferred to the glovebox. Thin films of **1-Th** and **1-U** were deposited by drop-casting a saturated THF solution of the compounds onto the slides. Optical microscopy measurements showed that the thin films completely bridged the channel between the gold electrodes (Figures S27-S30), suggesting formation of a connected network of particles. Current-voltage characteristics were measured with a BASi Epsilon-EC potentiostat under computer control using the DC potential amperometry function. The crocodile clip corresponding to the working electrode was connected to one electrode on one side of the slide, whereas those corresponding to the reference electrode and counter electrode were connected to an electrode soldered onto the opposite side of the slides. The voltages were set manually in the range between –3.2 V to +3.2 V. Voltages were applied for 10 seconds for each measurement with the glovebox temperature at 296 K.

The current (I) measured at individual voltages (V) were plotted to obtain the current-voltage characteristic curve. The resistance (R), was derived from a linear regression of the current-voltage curve following Ohm's first law, i.e., $I = V/R$ (eqn. S1), with the gradient being $1/R$.

The plates with the deposited films were then removed from the glovebox under an inert atmosphere and the area coverage estimated by optical microscopy. The thickness (t) of the film was measured via a stylus profilometer, and the conductivity (σ) was calculated with $\sigma = \frac{l}{Rwt}$ (eqn. S2), where l = 10 µm is the electrode channel length, w is the serpentine length along the interdigitated fingers (49.5 mm), t is the thickness of the thin film, and R is the resistance measured from the current-voltage curve.

For the low-temperature measurements, after the current-voltage curve was measured at room temperature, the slides were placed in the cold-well of the glovebox, which was cooled to 173 K with an external liquid nitrogen bath. The temperature was monitored with a low temperature thermometer. The slide was left for two hours in the cold well to ensure a stable temperature on the slide. The current-voltage curve was collected at 173 K following the methodology described above. To ensure that the quality of the film was not mechanically changed by the change in temperature, the samples were warmed again to room temperature and, after two hours, the conductivity was measured again. For both **1-Th** and **1-U**, the respective data are superimposable with the original room temperature measurements.

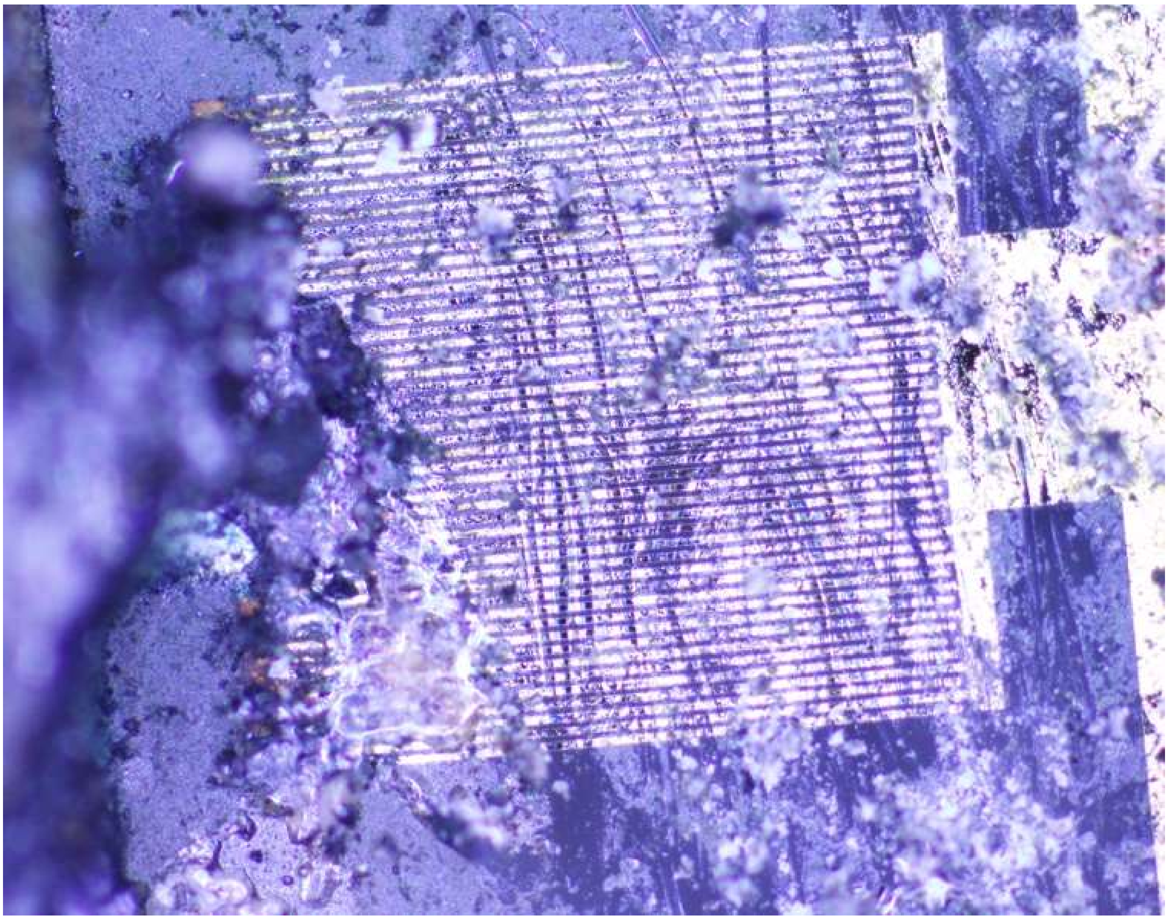

Figure S27. Optical microscope image of a thin film of **1-Th** on interdigitated gold electrodes.

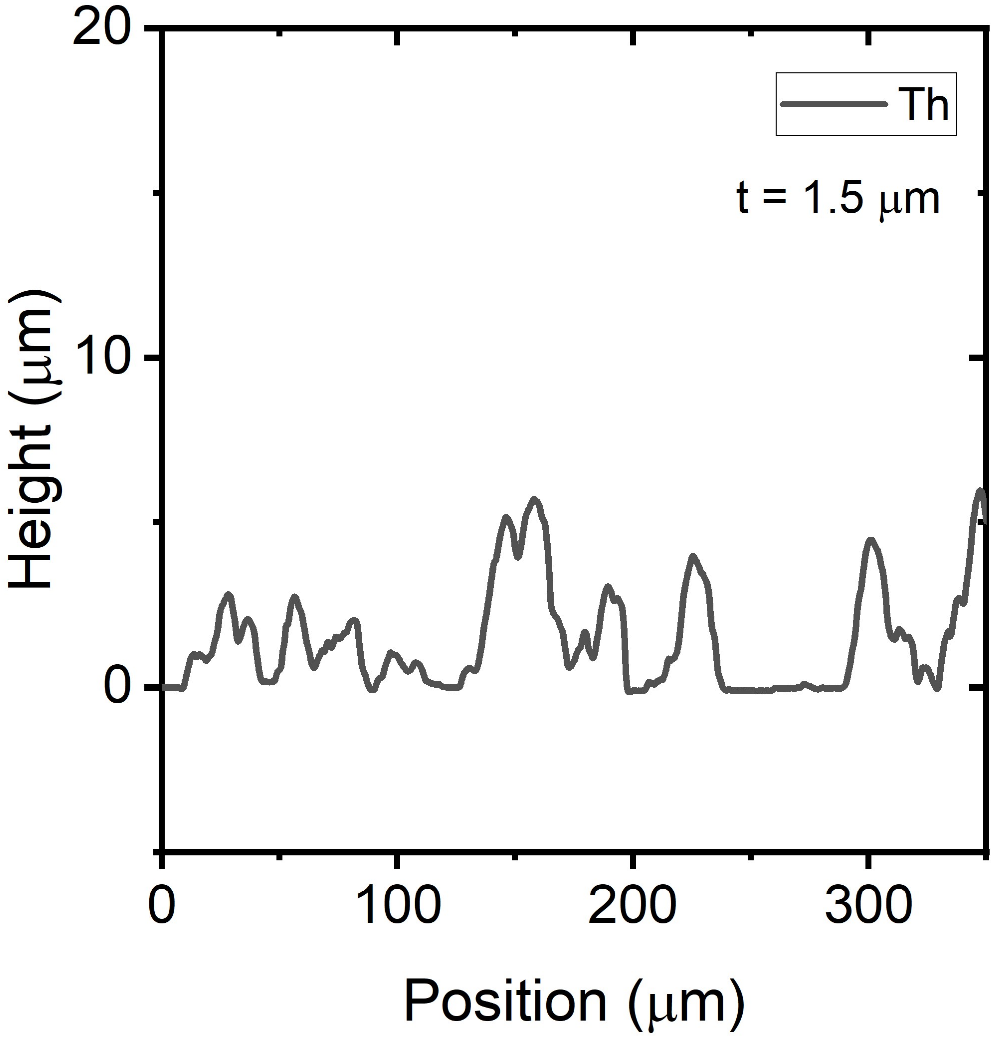

Figure S28. Profilometry data for a thin film of **1-Th**.

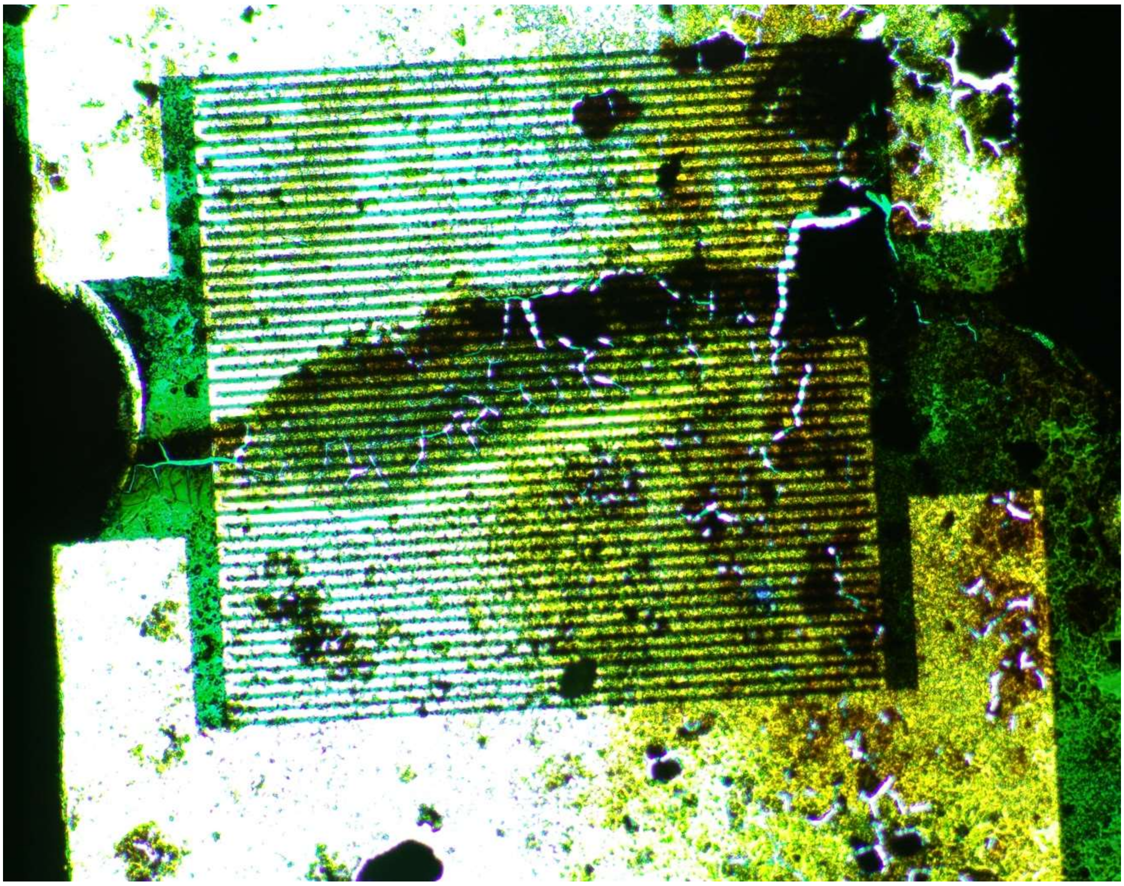

Figure S29. Optical microscope image of a thin film of **1-U** on interdigitated gold electrodes.

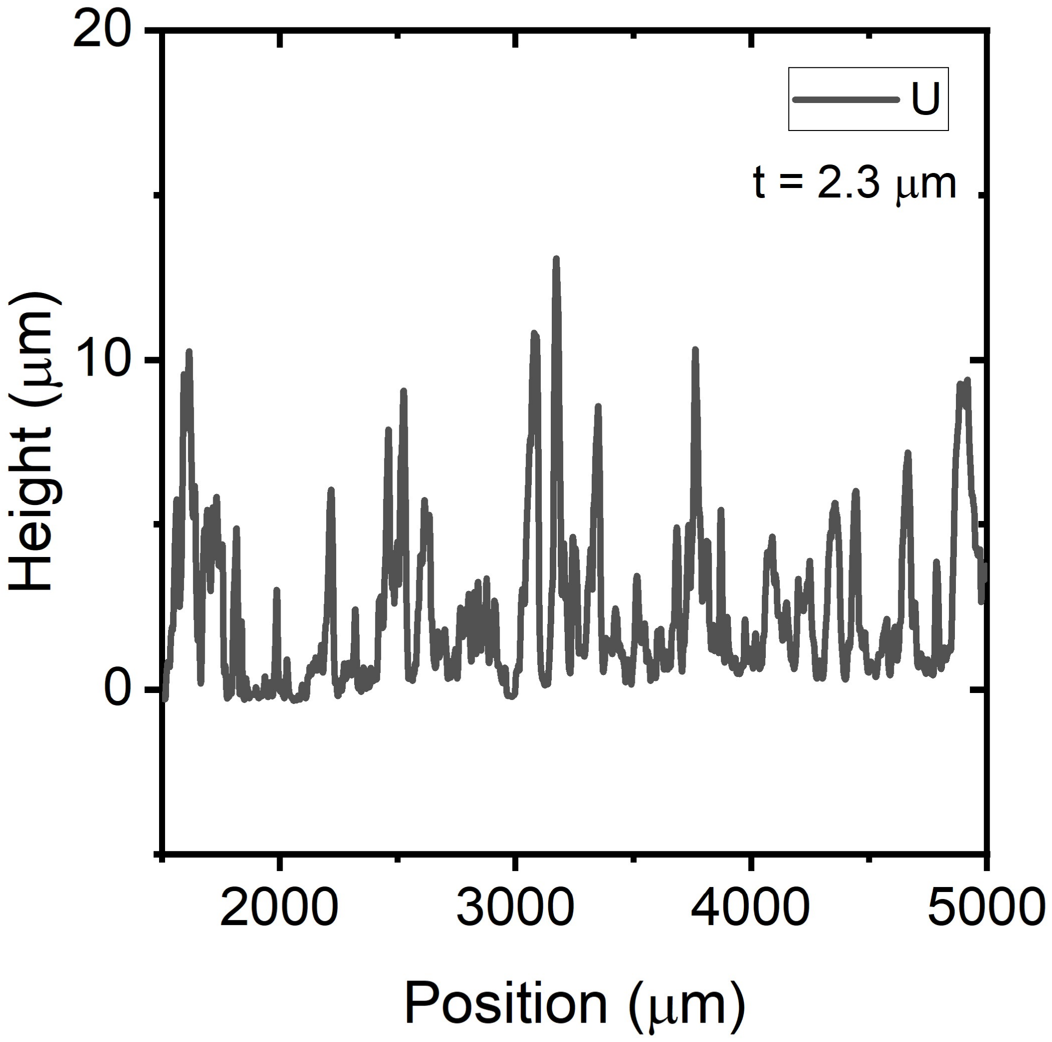

Figure S30. Profilometry data for a thin film of **1-U**.

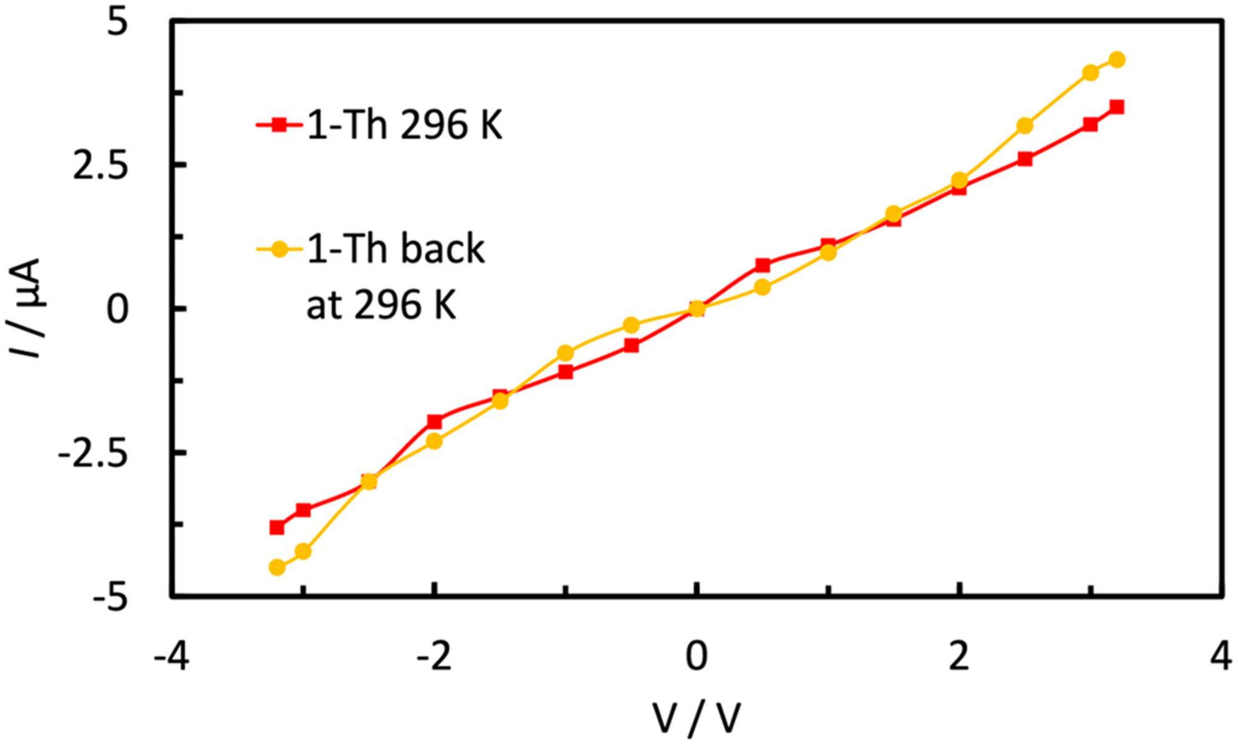

Figure S31. *I-V* data for **1-Th** measured initially at 296 K, and again at 296 K after cooling to 173 K.

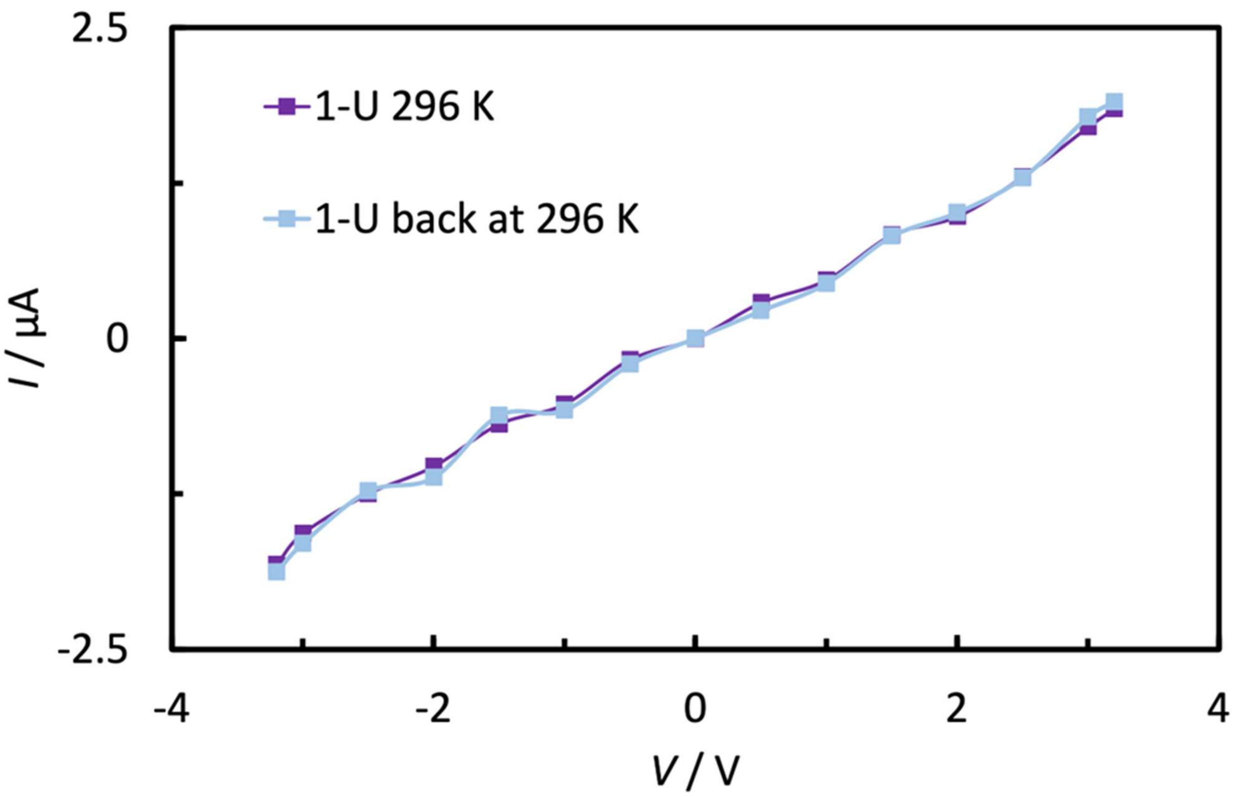

Figure S32. *I*-*V* data for **1-U** measured initially at 296 K, and again at 296 K after cooling to 173 K.

11. References

(1) Cantat, T.; Scott, B. L.; Kiplinger, J. L. Convenient Access to the Anhydrous Thorium Tetrachloride Complexes $ThCl_4(DME)_2$, $ThCl_4(1,4\text{-Dioxane})_2$ and $ThCl_4(THF)_{3.5}$ Using Commercially Available and Inexpensive Starting Materials. *Chem. Commun.* **2010**, *46*, 919–921.

(2) Wang, J.; Tee, K.; Lee, Y.; Riduan, S. N.; Zhang, Y. Hexaazatriphenylene Derivatives/GO Composites as Organic Cathodes for Lithium Ion Batteries. *J. Mater. Chem. A* **2018**, *6*, 2752–2757.

(3) Bergbreiter, D. E.; Killough, J. M. Reactions of Potassium-Graphite. *J. Am. Chem. Soc.* **1978**, *100*, 2126–2134.

(4) Khan, I. A.; Ahuja, H. S.; Bagnall, K. W.; Sinf, L. Uranium(IV) Chloride for Organometallic Synthesis. *Inorg. Synth.* **1982**, *43*, 187–190.

(5) Bain, G. A.; Berry, J. F. Diamagnetic Corrections and Pascal's Constants. *J. Chem. Educ.* **2008**, *85*, 532.

(6) Dolomanov, O. V; Bourhis, L. J.; Gildea, R. J.; Howard, J. A. K.; Puschmann, H. OLEX2: A Complete Structure Solution, Refinement and Analysis Program. *J. Appl. Crystallogr.* **2009**, *42*, 339–341.

(7) Sheldrick, G. M. A Short History of SHELX. *Acta Cryst. A* **2008**, *64*, 112–122.

(8) Sheldrick, G. M. Crystal Structure Refinement with SHELXL. *Acta Cryst. C* **2015**, *71*, 3–8.

(9) Ren, W.; Zi, G.; Walter, M. D. Synthesis, Structure, and Reactivity of a Thorium Metallocene Containing a 2,2′-Bipyridyl Ligand. *Organometallics* **2012**, *31*, 672–679.

(10) Zhang, L.; Hou, G.; Zi, G.; Ding, W.; Walter, M. D. Influence of the 5f Orbitals on the Bonding and Reactivity in Organoactinides: Experimental and Computational Studies on a Uranium Metallacyclopropene. *J. Am. Chem. Soc.* **2016**, *138*, 5130–5142.

(11) Frisch, M. J.; Trucks, G. W.; Schlegel, H. B.; Scuseria, G. E.; Robb, M. A.; Cheeseman, J. R.; Scalmani, G.; Barone, V.; Petersson, G. A.; Nakatsuji, H.; Li, X.; Caricato, M.; Marenich, A.; Bloino, J.; Janesko, B. G.; Gomperts, R.; Mennucci, B.; Hratchian, H. P.; Ortiz, J. V.; Izmaylov, A. F.; Sonnenberg, J. L.; Williams-Young, D.; Ding, F.; Lipparini, F.; Egidi, F.; Goings, J.; Peng, B.; Petrone, A.; Henderson, T.; Ranasinghe, D.; Zakrzewski, V. G.; Gao, J.; Rega, N.; Zheng, G.; Liang, W.; Hada, M.; Ehara, M.; Toyota, K.; Fukuda, R.; Hasegawa, J.; Ishida, M.; Nakajima, T.; Honda, Y.; Kitao, O.; Nakai, H.; Vreven, T.; Throssell, K.; Montgomery, J. A. Jr.; Peralta, J. E.; Ogliaro, F.; Bearpark, M.; Heyd, J. J.; Brothers, E.; Kudin, K. N.; Staroverov, V. N.; Keith, T.; Kobayashi, R.; Normand, J.; Raghavachari, K.; Rendell, A.; Burant, J. C.; Iyengar, S. S.; Tomasi, J.; Cossi, M.; Millam, J. M.; Klene, M.; Adamo, C.; Cammi, R.; Ochterski, J. W.; Martin, R. L.; Morokuma, K.; Farkas, O.; Foresman, J. B.; Fox, D. J. Gaussian 09. Gaussian, Inc.: Wallingford CT 2016.

(12) Yanai, T.; Tew, D. P.; Handy, N. C. A New Hybrid Exchange–Correlation Functional Using the Coulomb-Attenuating Method (CAM-B3LYP). *Chem. Phys. Lett.* **2004**, *393*, 51–57.

(13) Becke, A. D. Density-Functional Exchange-Energy Approximation with Correct Asymptotic Behavior. *Phys. Rev. A* **1988**, *38*, 3098–3100.

(14) Lee, C.; Yang, W.; Parr, R. G. Development of the Colle-Salvetti Correlation-Energy Formula into a Functional of the Electron Density. *Phys. Rev. B* **1988**, *37*, 785–789.

(15) Schäfer, A.; Horn, H.; Ahlrichs, R. Fully Optimized Contracted Gaussian Basis Sets for Atoms Li to Kr. *J. Chem. Phys.* **1992**, *97*, 2571–2577.

(16) Cao, X.; Dolg, M.; Stoll, H. Valence Basis Sets for Relativistic Energy-Consistent Small-Core Actinide Pseudopotentials. *J. Chem. Phys.* **2002**, *118*, 487–496.

(17) Küchle, W.; Dolg, M.; Stoll, H.; Preuss, H. Energy-adjusted Pseudopotentials for the Actinides. Parameter Sets and Test Calculations for Thorium and Thorium Monoxide. *J. Chem. Phys.* **1994**, *100*, 7535–7542.
(18) Eichkorn, K.; Weigend, F.; Treutler, O.; Ahlrichs, R. Auxiliary Basis Sets for Main Row Atoms and Transition Metals and Their Use to Approximate Coulomb Potentials. *Theor. Chem. Acc.* **1997**, *97*, 119–124.
(19) Weigend, F.; Häser, M.; Patzelt, H.; Ahlrichs, R. RI-MP2: Optimized Auxiliary Basis Sets and Demonstration of Efficiency. *Chem. Phys. Lett.* **1998**, *294*, 143–152.
(20) Seeger, R.; Pople, J. A. Self-consistent Molecular Orbital Methods. XVIII. Constraints and Stability in Hartree–Fock Theory. *J. Chem. Phys.* **2008**, *66*, 3045–3050.
(21) Bauernschmitt, R.; Ahlrichs, R. Stability Analysis for Solutions of the Closed Shell Kohn–Sham Equation. *J. Chem. Phys.* **1996**, *104*, 9047–9052.
(22) Noodleman, L. Valence Bond Description of Antiferromagnetic Coupling in Transition Metal Dimers. *J. Chem. Phys.* **1981**, *74*, 5737–5743.
(23) Jonkers, G.; de Lange, C. A.; Noodleman, L.; Baerends, E. J. Broken Symmetry Effects in the He(I) Valence Photoelectron Spectrum of $Se(CN)_2$. *Mol. Phys.* **1982**, *46*, 609–620.
(24) Noodleman, L.; Norman, J. G. Jr.; Osborne, J. H.; Aizman, A.; Case, D. A. Models for Ferredoxins: Electronic Structures of Iron-Sulfur Clusters with One, Two, and Four Iron Atoms. *J. Am. Chem. Soc.* **1985**, *107*, 3418–3426.
(25) Noodleman, L.; Davidson, E. R. Ligand Spin Polarization and Antiferromagnetic Coupling in Transition Metal Dimers. *Chem. Phys.* **1986**, *109*, 131–143.
(26) Moreira, I. de P. R.; Illas, F. A Unified View of the Theoretical Description of Magnetic Coupling in Molecular Chemistry and Solid State Physics. *Phys. Chem. Chem. Phys.* **2006**, *8*, 1645–1659.
(27) Yamaguchi, K.; Fukui, H.; Fueno, T. Molecular Orbital (MO) Theory For Magnetically Interacting Organic Compounds. Ab-Initio Mo Calculations of the Effective Exchange Integrals for Cyclophane-Type Carbene Dimers. *Chem. Lett.* **1986**, *15*, 625–628.
(28) Yamaguchi, K.; Tsunekawa, T.; Toyoda, Y.; Fueno, T. Ab Initio Molecular Orbital Calculations of Effective Exchange Integrals between Transition Metal Ions. *Chem. Phys. Lett.* **1988**, *143*, 371–376.
(29) Yamaguchi, K.; Jensen, F.; Dorigo, A.; Houk, K. N. A Spin Correction Procedure for Unrestricted Hartree-Fock and Møller-Plesset Wavefunctions for Singlet Diradicals and Polyradicals. *Chem. Phys. Lett.* **1988**, *149*, 537–542.
(30) te Velde, G.; Bickelhaupt, F. M.; Baerends, E. J.; Fonseca Guerra, C.; van Gisbergen, S. J. A.; Snijders, J. G.; Ziegler, T. Chemistry with ADF. *J. Comput. Chem.* **2001**, *22*, 931–967.
(31) Fonseca Guerra, C.; Snijders, J. G.; te Velde, G.; Baerends, E. J. Towards an Order-N DFT Method. *Theor. Chem. Acc.* **1998**, *99*, 391–403.
(32) Kitaura, K.; Morokuma, K. A New Energy Decomposition Scheme for Molecular Interactions within the Hartree-Fock Approximation. *Int. J. Quantum Chem.* **1976**, *10*, 325–340.
(33) Ziegler, T.; Rauk, A. On the Calculation of Bonding Energies by the Hartree Fock Slater Method. *Theor. Chim. Acta* **1977**, *46*, 1–10.
(34) Ziegler, T.; Rauk, A. A Theoretical Study of the Ethylene-Metal Bond in Complexes between Copper(1+), Silver(1+), Gold(1+), Platinum(0) or Platinum(2+) and Ethylene, Based on the Hartree-Fock-Slater Transition-State Method. *Inorg. Chem.* **1979**, *18*, 1558–1565.
(35) Grimme, S.; Ehrlich, S.; Goerigk, L. Effect of the Damping Function in Dispersion Corrected Density Functional Theory. *J. Comput. Chem.* **2011**, *32*, 1456–1465.

(36) Grimme, S.; Antony, J.; Ehrlich, S.; Krieg, H. A Consistent and Accurate Ab Initio Parametrization of Density Functional Dispersion Correction (DFT-D) for the 94 Elements H-Pu. *J. Chem. Phys.* **2010**, *132*, 154104.
(37) van Lenthe, E.; Baerends, E. J.; Snijders, J. G. Relativistic Regular Two-component Hamiltonians. *J. Chem. Phys.* **1993**, *99*, 4597–4610.
(38) van Lenthe, E.; Baerends, E. J.; Snijders, J. G. Relativistic Total Energy Using Regular Approximations. *J. Chem. Phys.* **1994**, *101*, 9783–9792.
(39) van Lenthe, E.; van Leeuwen, R.; Baerends, E. J.; Snijders, J. G. Relativistic Regular Two-Component Hamiltonians. *Int. J. Quantum Chem.* **1996**, *57*, 281–293.
(40) van Lenthe, E.; Baerends, E. J. Optimized Slater-Type Basis Sets for the Elements 1–118. *J. Comput. Chem.* **2003**, *24*, 1142–1156.
(41) van Gisbergen, S. J. A.; Snijders, J. G.; Baerends, E. J. Implementation of Time-Dependent Density Functional Response Equations. *Comput. Phys. Commun.* **1999**, *118*, 119–138.
(42) Akinaga, Y.; Ten-no, S. Range-Separation by the Yukawa Potential in Long-Range Corrected Density Functional Theory with Gaussian-Type Basis Functions. *Chem. Phys. Lett.* **2008**, *462*, 348–351.
(43) Seth, M.; Ziegler, T.; Steinmetz, M.; Grimme, S. Modeling Transition Metal Reactions with Range-Separated Functionals. *J. Chem. Theory. Comput.* **2013**, *9*, 2286–2299.
(44) Klamt, A.; Jonas, V. Treatment of the Outlying Charge in Continuum Solvation Models. *J. Chem. Phys.* **1996**, *105*, 9972–9981.
(45) Klamt, A.; Schüürmann, G. COSMO: A New Approach to Dielectric Screening in Solvents with Explicit Expressions for the Screening Energy and Its Gradient. *J. Chem. Soc., Perkin Trans. 2* **1993**, 799–805.
(46) Klamt, A. Conductor-like Screening Model for Real Solvents: A New Approach to the Quantitative Calculation of Solvation Phenomena. *J. Phys. Chem.* **1995**, *99*, 2224–2235.
(47) Stoll, S.; Schweiger, A. EasySpin, a Comprehensive Software Package for Spectral Simulation and Analysis in EPR. *J. Magn. Res.* **2006**, *178*, 42–55.
